# Addressing the Osteoporosis Problem—Multifunctional Injectable Hybrid Materials for Controlling Local Bone Tissue Remodeling

**DOI:** 10.1021/acsami.1c17472

**Published:** 2021-10-13

**Authors:** Adriana Gilarska, Alicja Hinz, Monika Bzowska, Grzegorz Dyduch, Kamil Kamiński, Maria Nowakowska, Joanna Lewandowska-Łańcucka

**Affiliations:** † Faculty of Chemistry, Jagiellonian University, Gronostajowa 2, 30-387 Kraków, Poland; ‡ Faculty of Physics and Applied Computer Science, AGH University of Science and Technology, Mickiewicza 30, 30-059 Kraków, Poland; § Department of Cell Biochemistry, Faculty of Biochemistry, Biophysics and Biotechnology Jagiellonian University, Gronostajowa 7, 30-387 Kraków, Poland; ∥ Department of Pathomorphology, Jagiellonian University Medical College, 30-387 Kraków, Poland

**Keywords:** biopolymers, injectable hydrogels, silica−apatite-based alendronate carriers, biocompatibility in vivo, osteoporosis, bone tissue engineering

## Abstract

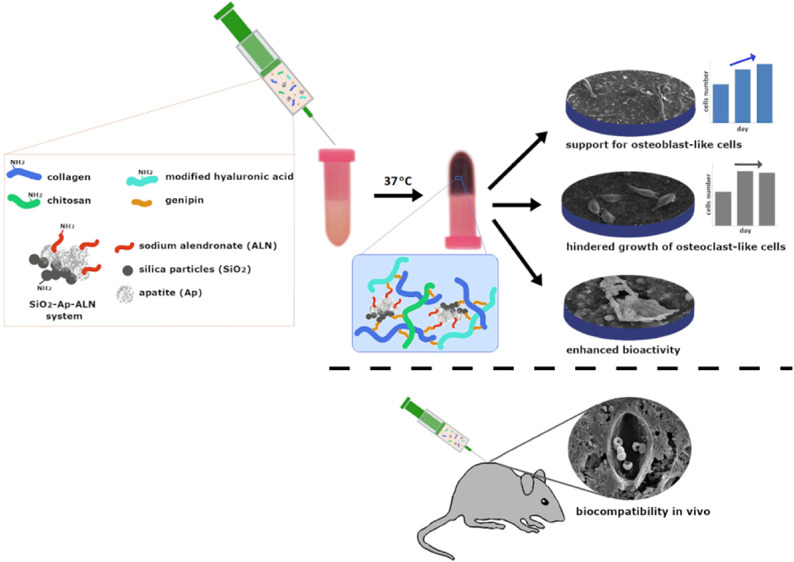

Novel multifunctional biomimetic injectable hybrid systems were synthesized. The physicochemical as well as biological in vitro and in vivo tests demonstrated that they are promising candidates for bone tissue regeneration. The hybrids are composed of a biopolymeric collagen/chitosan/hyaluronic acid matrix and amine group-functionalized silica particles decorated with apatite to which the alendronate molecules were coordinated. The components of these systems were integrated and stabilized by cross-linking with genipin, a compound of natural origin. They can be precisely injected into the diseased tissue in the form of a viscous sol or a partially cross-linked hydrogel, where they can serve as scaffolds for locally controlled bone tissue regeneration/remodeling by supporting the osteoblast formation/proliferation and maintaining the optimal osteoclast level. These materials lack systemic toxicity. They can be particularly useful for the repair of small osteoporotic bone defects.

## 1 Introduction

Osteoporosis is one of the most progressive, systemic, and metabolic diseases affecting the bone tissue. Causing the reduction of bone mass and microstructural bone deterioration, it results in more than 9 million fractures annually worldwide.^1^ Moreover, the incidence of osteoporosis is increasing with the aging populations in developed countries. Its origin relies on the imbalance between bone tissue formation (governed by osteoblasts) and bone tissue absorption (controlled by osteoclasts) that occur during bone remodeling.^2^ When the bone formation is slower than bone resorption, each remodeling event removes a small part of bone from the skeleton and leads to bone loss and structural damage, which further enhances the bone turnover (more resorptive cavities on a given surface at a given time and a shorter mineralization time of bones).^3,4^ According to the World Health Organization, older people, especially postmenopausal women experience acceleration in bone loss, making osteoporosis a serious public health problem in the world.^5^ For that reason, timely prevention and appropriate therapy are of principal importance.

Current osteoporosis treatments are limited mainly to anabolic agents and antiresorptive drugs.^6^ Antiresorptive compounds [e.g., bisphosphonates (BPs)] restore bone strength by suppressing the rate of bone remodeling, promoting the completion of bone formation, and reducing the depth of resorption in each of the reduced number of bone metabolic units engaged in the remodeling process. Alendronate (ALN), a bone resorption inhibitor, is the most commonly prescribed oral nitrogen-containing BP for the therapy of postmenopausal osteoporosis, corticosteroid-induced osteoporosis, Paget’s disease, and metastatic bone diseases. The nitrogen groups present within the R2 side group are associated with the ALN ability to inhibit farnesyl pyrophosphate synthase, a major enzyme in the mevalonate pathway. As a result, the activities of the osteoclasts are disrupted, including the migration, attachment, and resorption, and, ultimately, cell death can occur via apoptosis.^7^ However, being a BCS III drug (high solubility and low permeability due to its polar hydrophilic nature), ALN is characterized by limited oral absorption and bioavailability (less than 1%).

Furthermore, ALN administration is associated with serious side effects, including jaw osteonecrosis, irritation of the gastrointestinal system, nausea, musculoskeletal pain, and cardiovascular risks. Also, the intravenous administration of ALN carries the risk of nephrotoxicity, fever and flu symptoms, as well as electrolyte imbalance.^8^ These issues might be addressed by delivering the antiosteoporotic agents specifically to the diseased bone tissues. Therefore, systems enabling local administration and thus a localized action of drugs seem to be extremely attractive, ensuring bone resorption limitation while restricting the systemic side effects during the whole therapy. Various types of ALN delivery systems are reported in the literature including micro- and nanoformulations, mesoporous materials, and hydrogel-based materials, as well as calcium phosphate cement.^8−10^ However, it was found that ALN was quite easily lost in the aqueous phase during encapsulation and quickly diffused out from the carrier, thereby resulting in lower drug encapsulation efficiency and a greater initial burst release that may significantly increase the risk of tissue irritation or damage. Also, due to the intrinsic permeability and limited network interactions with small-molecule drugs within the hydrogel matrix, sustained delivery by means of such a macromolecular formulation is still challenging. A novel ALN delivery system proposed in this paper is based on hydroxyapatite (HAp)-decorated silica-based particles appropriately functionalized to ensure their covalent attachment to a hydrogel matrix on a cross-linking process. Hap is a bone-mimetic material that possesses mechanical properties resembling the native bone tissues. HAp is frequently clinically used as a bioactive and biocompatible material to support the biointegration of implants of both hard and soft tissue. It was found that ALN exhibits affinity to HAp,^11^ specifically, the deprotonated oxygen atoms of the phosphate groups of ALN interact electrostatically with Ca^2+^ cations of Hap. In addition to acting as drug carriers, HAp can also serve as a reservoir for calcium and phosphate ions, necessary for bone homeostasis and mineral formation under physiological conditions, which is attributed to its osteogenic and osteoinductive properties.^12^ Herein, amine group-functionalized silica particles decorated with HAp under biomimetic simulated body fluid (SBF) conditions and attached with ALN utilizing the ALN–HAp affinity are presented. It was established that silica-based particles exhibit strong biological activities and thus might be promising drug carriers.^13^ In addition, the use of amine-functionalized silica particles enables the deposition of HAp to be controlled without the need of complicated procedures. However, pristine HAp-decorated silica particles will not ensure the local delivery of the carried drugs. In order to overcome these limitations, a novel approach based on the fabrication of hybrids by introducing carriers in the form of functionalized silica particles decorated with HAp into the biopolymeric hydrogel matrix is proposed within this work.

Polymeric hydrogels are preferred materials for the preparation of various systems for bone tissue engineering applications due to their interesting physical properties that make them similar to natural tissues. Hydrogels of properly selected chemical composition allow the preparation of materials characterized by both biocompatibility and increased structural integrity, so that cell adhesion, growth, and differentiation are ensured.^14^ Moreover, by using the injectable hydrogels, their introduction into tissue defects in a relatively non-invasive way and precise filling of the existing bone cavity are possible. The selection of biopolymers for hydrogels proposed in this paper was dictated by the idea of mimicking the composition of the natural extracellular matrix (ECM) in order to facilitate cell attachment, proliferation, and differentiation. Thus, the antiresorptive activity of SiO_2_–Ap–ALN will be simultaneously supported by new bone formation. For these reasons, native-like and abundant biopolymers with inherent bioactivity have been taken into account, namely, collagen (Col), lysine-functionalized hyaluronic acid (HAmod), and chitosan (Ch). Therefore, in this work, we present a novel multifunctional delivery system that seems to represent an extremely promising alternative to the formulations investigated so far. The developed materials will simultaneously possess antiosteoporotic (ALN) properties and mimic the architecture and chemical composition of the natural bone tissue (biopolymeric matrix reinforced with apatite (Ap)-decorated silica particles), thus facilitating bone regeneration and biointegration while displaying desired physicochemical features (mechanical properties, wettability, and swellability).

Considering the potential future applications of the developed materials, the in vivo biological evaluation using a mouse model was also performed. The injectability, as well as the ability to gel in vivo, was verified. Moreover, a panel of biochemical and histopathological analyses enabled the determination of hemo-, hepato-, or nephrotoxicity. These will allow a reliable assessment of the biocompatibility of the developed biomaterials.

## 2 Materials and Methods

### 2.1 Materials

See the Supporting Information.

### 2.2 Sample Preparation

#### 2.2.1 SiO_2_–Ap Particle Preparation

Silica particles were obtained according to the procedure described in our previous paper.^15^ Deposition of Ap on the surface of silica particles was carried out using SBF (1.5 SBF) prepared according to Kokubo’s method.^16^ For that purpose, 20 mg of SiO_2_ particles was added to 20 mL of 1.5 M SBF solution. The samples were sonicated continuously for 10–15 min and then incubated at 37 °C with gentle shaking (50 rpm). Incubation was carried out for 10 days, and the SBF solution was replaced every 2–3 days. Then, the material was centrifuged, rinsed with water (procedure repeated three times), and dried at room temperature. A white powder SiO_2_–Ap material was obtained.

#### 2.2.2 SiO_2_–Ap–ALN Particle Preparation

Sodium ALN was attached to the obtained SiO_2_–Ap system. For this purpose, 20 mg of SiO_2_–Ap material was suspended in 3 mL of 5 mM NaOH and sonicated for 5 min. Then, 4 mg of ALN was dissolved in 2 mL of 5 mM NaOH. The pH of the ALN solution was adjusted to 10 by adding 20 mM NaOH. Then ALN solution was added to the SiO_2_–Ap suspension. The sample was placed on a magnetic stirrer with heating (500 rpm, 37 °C) for 3 days. The sodium ALN carrier (SiO_2_–Ap–ALN) was purified by dialysis (24 h, room temperature) and lyophilized, and a white SiO_2_–Ap–ALN powder was obtained.

#### 2.2.3 Preparation of Hydrogels and Hybrid Materials

A series of SiO_2_–Ap–ALN samples were prepared by suspending 5 mg of C1, 2.5 mg of C2, and 1 mg of C3 in 0.1 mL of water separately. Then, appropriate amounts of biopolymer solutions were added to each sample: 76 μL of Ch solution (1 wt % in 1% acetic acid), 540 μL of Col solution, and 114 μL of HAmod solution [1 wt % in 10× phosphate-buffered saline (PBS)]. The mixtures were vigorously vortexed, then 170 μL of genipin solution (20 mM in 10× PBS) was added to each sample and incubated at 37 °C until the gel formation was complete. The Col/Ch/HAmod weight ratio in the hydrogels prepared was 50:20:30. Using three different concentrations of SiO_2_–Ap–ALN dispersions [5 mg/mL (C1), 2.5 mg/mL (C2), and 1 mg/mL (C3)], three types of hybrid materials were obtained: ColChHA_mod_ C1, ColChHA_mod_ C2, and ColChHA_mod_ C3. A hydrogel of analogous biopolymer composition (ColChHA_mod_) but without the SiO_2_–Ap–ALN particles was prepared as a control material.

### 2.3 Methods

Characterization details of silica–Ap (SiO_2_–Ap), silica–Ap–ALN (SiO_2_–Ap–ALN) particles, and hybrid materials are presented in the Supporting Information.

#### 2.3.1 Evaluation of the ALN Content in SiO_2_–Ap–ALN Particles

The amounts of ALN immobilized in the SiO_2_–Ap–ALN particles were evaluated using a spectrophotometric assay based on the formation of a color complex between ALN and Fe^3+^ ions in the presence of perchloric acid.^17^ Briefly, 0.5 mL of SiO_2_–Ap–ALN dispersion (1 mg/mL in 0.2 M perchloric acid) was mixed with 0.15 mL of 5 mM FeCl_3_ solution in perchloric acid (0.2 M), and the volume was made up to 3 mL with perchloric acid (0.2 M). Absorbance was measured at 300 nm using the SiO_2_–Ap dispersion in perchloric acid as a blank. The ALN content was determined based on the calibration curve of ALN in 0.2 M perchloric acid in the concentration range of 4–63 μg/mL. The spectrophotometric determination of the ALN content in the developed particles was about 6%. The weight content of immobilized ALN was also calculated based on the increase of the final weight loss in thermogravimetric (TG) profiles measured in an inert atmosphere for SiO_2_–Ap and SiO_2_–Ap–ALN particles, respectively. This value was about 3.3%.

#### 2.3.2 Drug Release Studies

Hybrid materials with the highest concentration of the SiO_2_–Ap–ALN system (ColChHA_mod_ C1) were immersed in 1 mL of PBS and incubated at 37 °C with shaking at 100 rpm. At certain time points (after 1, 2, 4, 6, 24, 48, 72, 144, and 168 h), all the release media were collected and replaced with fresh PBS. The same procedure was used for drug-free hybrid materials that were used as the reference. Collected samples were stored at −20 °C until analysis. The spectrophotometric method based on the formation of a complex of ALN and Fe(III) ions was used to determine the amount of released drug. For each sample, the experiments were carried out in triplicates, and the results were presented as the average values. Additionally, the experimental data were fitted to three kinetic models for analyzing the release mechanism: Higuchi, Ritger–Peppas, and Weibull models.

#### 2.3.3 In Vitro Biomineralization

In vitro biomineralization studies were performed in SBF prepared according to Kokubo’s method.^16^ For more details, please see the Supporting Information.

#### 2.3.4 Biological Experiments In Vitro

Details of in vitro biological experiments using osteoblast-like (MG-63) and osteoclast-like (J774A.1) cells and the preparation of the materials for cell culture are provided in the Supporting Information.

#### 2.3.5 Alamar Blue Cell Viability and Alkaline Phosphatase Assays

After 1, 3, and 7 days of culture, the cell viability was studied using the Alamar Blue (AB) assay according to the procedure employed by us previously.^18^ Based on the standard curves established with MG-63 or J774A.1 cells cultured at various densities, the cell number was calculated. The alkaline phosphatase (ALP) activity of MG-63 cells was studied at culture days 3 and 7 using the protocol described by us earlier.^18^

#### 2.3.6 Cell Morphology and Adhesion Study

After 3 days of MG-63 culturing, the tested materials were fixed and dehydrated using a protocol developed by us earlier.^19^ Microscopic observations were performed employing a cold field-emission scanning electron microscope (Hitachi S-4700).

#### 2.3.7 Biological Experiments In Vivo

Details of in vivo tests are provided in the Supporting Information.

#### 2.3.8 Analysis of Material Injection, Biodegradability, and Biocompatibility

C57BL/6 mice were weighed and randomly divided into experimental groups (a control group—injected with sterile PBS, a group injected with ColChHAmod, and a group injected with ColChHAmod C1). Prior to experiments, materials to be tested were prepared by mixing all compounds with genipin solution (20 mM in 10× PBS), transferred into a syringe, and incubated at 37 °C for 15 min to induce gel formation. Next, mice were injected subcutaneously (using a 29G needle) with 200 μL of materials into the left flank. The animals were examined after material administration every day for 7 days to detect changes in the size and the volume of materials visible under the skin (palpable examination and measurements with a caliper). After the first week, mice were observed at 7 day intervals. Mice were euthanized after 24 h (the first day of the experiment), 7, 30, and 60 days after material administration. Blood, main organs, and skin fragments with the remaining hydrogel debris were isolated for further analyses. The volume of ColChHAmod C1 was calculated according to the following formula
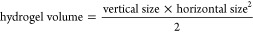
1

#### 2.3.9 Blood Morphology Analyses and Serum Biochemical and Immunological Analyses

On the day of euthanasia, blood was first taken from the facial vein for hematological analyses performed with a fully automated veterinary analyzer ABC Vet (HORIBA, UK): counts of white blood cells, red blood cells, platelets, granulocytes, lymphocytes (LYM), and monocytes; hematocrit value, mean corpuscular volume; hemoglobin concentration; and red blood cell distribution width. Next, blood was drawn postmortem by cardiac puncture, allowed to clot, and centrifuged (800*g*, 10 min, RT). Serum isolated by centrifugation (800*g*, 10 min, RT) was used for biochemical analysis (using a SPOTCHEM EZ Chemistry Analyzer—Woodley Equipment and multiparameter strips SPOTCHEM II Panel V, according to the manufacturer’s instructions) and cytokine profiling. The levels of IL-1α, IL-1β, IL-6, IL-10, IL-12p70, IL-17α, IL-23, IL-27, MCP-1, IFN-β, IFN-γ, TNF-α, and GM-CSF were examined using the LEGENDplex Mouse Inflammation Panel (13-plex) immunoassay (BioLegend) and a BD LSRFortessa flow cytometer, and analyzed using LEGENDplex software (BioLegend).

#### 2.3.10 Tissue Processing and Staining for Histological Examination

Isolated skins with hydrogel fragments, liver, kidney, and spleen were fixed. Briefly, the tissues were incubated in formalin stored at room temperature for 24 h. The tissues were then placed for several days at 4 °C to carry out the dehydration process. The tissues were transferred to the described histological cages the day before dehydration and placed in 70% EtOH. The next day, a series of incubations were carried out in solutions with increasing concentrations of EtOH, xylene, and finally in dissolved paraffin (60 °C) according to the protocol proposed by Berry et al.^100^ After overnight incubation in dissolved paraffin, tissue fragments were sunk in paraffin blocks using the HistoStar sinking station (Thermo Scientific). Subsequently, tissue sections of 4 μm thickness were obtained using a microtome (Thermo Scientific), which were transferred to ready-made poly-l-lysine (Thermo Scientific) primary slides. Before staining, the preparations were subjected to paraffin removal by incubation at 60 °C for 60 min, followed by 5 min of incubation in xylene solution three times, and 5 min of incubation in 99.8% ethyl alcohol solution (EtOH) three times. Subsequently, the tissues were hydrated by incubation in EtOH solutions (95% and then 80%) and finally in water for 2 min. After deparaffinization, the preparations were stained either with hematoxylin and eosin or Masson’s trichrome stain according to the manufacturer’s recommendations (Sigma-Aldrich Kit). The specimens were sealed with the Cytoseal XYL Thermo Scientific mounting medium and examined with the Leica DM6B fluorescence microscope under bright field at 5-, 10-, or 20× magnification.

#### 2.3.11 Staining of Hydrogel Fragments Isolated from the Skin with Alizarin Red

Isolated skins with hydrogel fragments were fixed in the same way as described above for tissue processing and staining for histological examination. The preparations were then stained with a solution of 2% Alizarin red in distilled water for 2 min, and then they were dehydrated by 20 times (20 dips) immersion in acetone, followed by 20 times (20 dips) immersion in an acetone/xylene solution mixed in a ratio of 1:1. The preparations were then incubated for 1 min in xylene and eventually transferred to fresh xylene before sealing. The specimens were sealed with the Cytoseal XYL Thermo Scientific mounting medium and examined with the Leica DM6B fluorescence microscope under bright field at 5× magnification.

### 2.4 Statistical Analysis

Experiments were repeated three times, and the results were expressed as mean ± standard deviation. Statistical significance was calculated using the one-way analysis of variance (ANOVA) with Tukey’s honestly significant difference post hoc test (swelling) and Student’s t-*t*est (degradation, rheological measurements, proliferation and ALP activity studies, and cell spreading). A comparison between two means was made with a statistical significance level set at *p* < 0.05.

## 3 Results and Discussion

### 3.1 Sodium ALN Carrier in the Form of Hybrid System SiO_2_–Ap–ALN: Fabrication and Physicochemical Characterization

**Figure 1:**
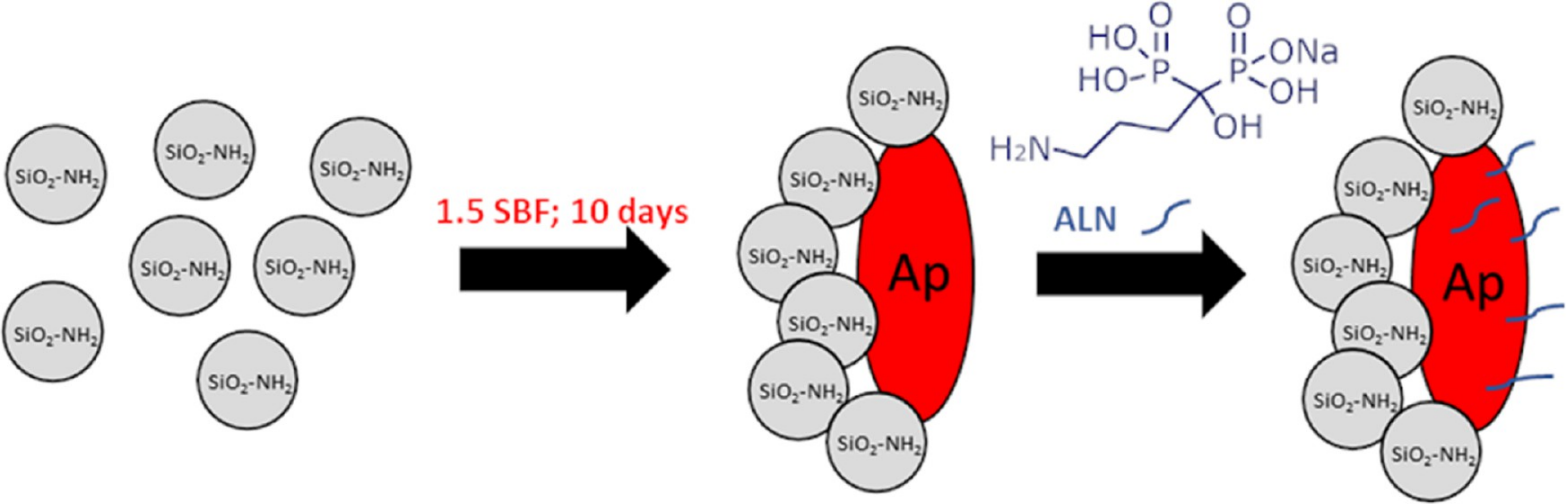
Scheme of SiO_2_–Ap–ALN particle synthesis (SiO_2_–NH_2_—silica particles functionalized with amine groups; Ap—apatite formed on the surface of silica particles; and ALN—sodium alendronate).

A sodium ALN carrier in the form of SiO_2_–Ap–ALN particles was fabricated in a two-step procedure, as schematically depicted in Figure 1. In the first step, SiO_2_–Ap particles were obtained. The controlled Ap deposition on the amine-functionalized silica particles was carried out according to the procedure presented by us previously^15^ under SBF conditions, ensuring similar inorganic ion concentrations to those characterizing the human plasma.^16^ That approach based on biomimetic mineralization is attractive and extremely useful because it proceeds under mild conditions. It has been established that silanol groups (Si–OH) present on the surface of silica particles play a crucial role in nucleation of Ap formed. Under the SBF conditions, the Si–OH groups have a negative charge (Si–O^–^). Thus, Si–O^–^ can interact with positively charged Ca^2+^ ions to form a Ca-rich positively charged thin layer, which in turn combines with negatively charged PO_4_^3–^ ions resulting in the creation of amorphous calcium phosphate that eventually transforms into Ap in SBF.^20^ Moreover, it was confirmed that the adsorption of phosphate groups on amino group-terminated surfaces may also induce Ap formation (by the PO_4_^3–^–NH^3+^ ion–ion interaction).^21^

**Figure 2:**
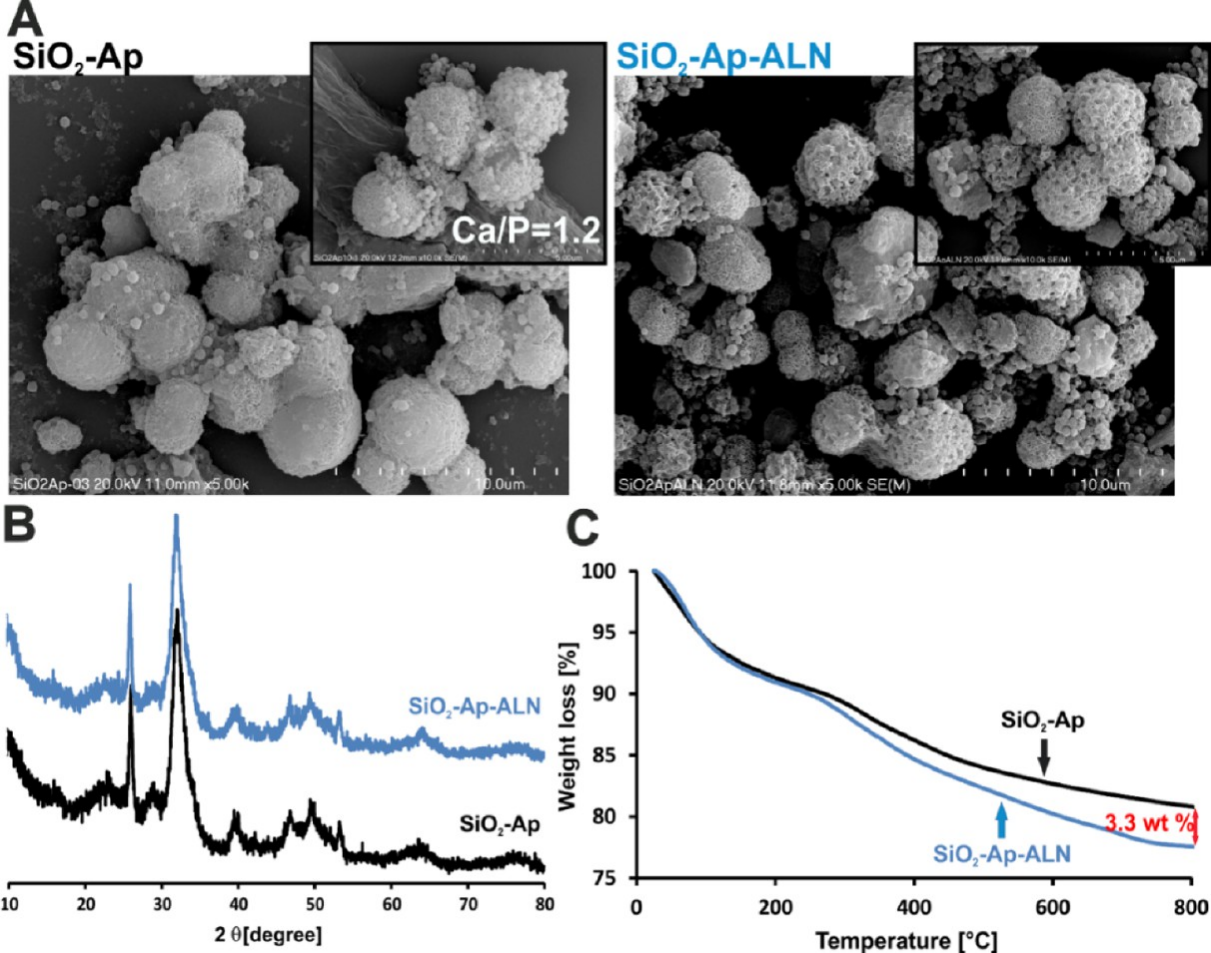
(A) SEM micrographs, (B) XRD pattern, and (C) TG profiles obtained in an inert atmosphere (argon) for silica particles covered with Ap (SiO_2_–Ap) and ALN-attached (SiO_2_–Ap–ALN).

Scanning electron microscopy (SEM) and energy-dispersive X-ray spectroscopy (EDS) investigation of SiO_2_–Ap particles demonstrated the formation of mineral phases with a morphology and a Ca/P ratio characteristic of Ap-like structures (see Figure 2A). Moreover, X-ray diffraction (XRD) analysis confirmed unambiguously the presence of a crystalline phase in the obtained materials. In the XRD pattern depicted in Figure 2B, the diffraction peaks at 2θ: 25.9; 32.0; 39.4; 42.2; 46.8, and 53.2 are in accordance with signals ascribed to Ap-like structures reported in the literature.^11^ Overall, it might be concluded that the proposed methodology allows for the successful synthesis of bioactive SiO_2_–Ap particles under mild conditions stimulating the biomineralization process.

In the next step, sodium ALN was attached to SiO_2_–Ap particles utilizing its affinity to HAp. For that purpose, the procedure developed by Ma et al. was employed.^11^ The stability of the obtained material is ensured by the interaction resulting from strong ALN affinity to HAp. It was found that deprotonated oxygen atoms of the phosphate groups of ALN interact electrostatically with Ca^2+^ cations of Ap.^12^ The SiO_2_–Ap–ALN particles formed in that process were characterized using a series of characterization methods: SEM, X-ray photoelectron spectroscopy (XPS), XRD, Fourier-transform infrared (FTIR) spectroscopy, and TG techniques. The content of immobilized ALN was evaluated using the spectrophotometric assay.

The FTIR spectra of SiO_2_, SiO_2_–Ap, SiO_2_–Ap–ALN, and ALN are shown in Figure S1 (Supporting Information). As can be seen in Figure S1, SiO_2_, SiO_2_–Ap, and SiO_2_–Ap–ALN systems show typical peaks related to the structure of silica particles—a strong peak at 1020 cm^–1^ as well as that at 779 cm^–1^ can be assigned to the Si–O–Si bonds and a peak at 959 cm^–1^ can be attributed to the SiOH groups. Additionally, a peak at 1540 cm^–1^ can be assigned to the NH_2_ groups, which were associated with the APTES skeleton.^15^ For SiO_2_–Ap and SiO_2_–Ap–ALN systems, a band at 1450 cm^–1^ and a weak peak at 877 cm^–1^ appeared, which can be attributed to the CO_3_^2–^ groups from the Ap structures (black arrows).^22^ Characteristic peaks for ALN are in the range 1200–900 cm^–1^, which correspond to C–O and P=O stretches.^23^ However, it is not possible to distinguish these peaks in the spectrum of the SiO_2_–Ap–ALN system, because they coincide with the characteristic range of bands for both silica and HAp.

To gain more insights into the chemical composition of the fabricated materials, XPS analyses were performed. Because the peak intensities in the XPS spectra are directly related to the atomic distribution of material’s surface, they can be used to quantify the composition of atomic elements.^24^ The XPS spectra of SiO_2_–Ap and SiO_2_–Ap–ALN are depicted in Figure S2 (Supporting Information), while the atomic compositions (%) of the surface of the obtained particles are presented in Table S1. The peaks observed can be assigned to O 1s (533 eV), N 1s (401 eV), Ca 2p (349 eV), C 1s (286 eV), P 2p (134 eV), and Si 2p (105 eV), respectively.^25^ The analysis of the surface composition of materials developed (see Table S1) indicated that N 1s and C 1s signals increased 2-fold for SiO_2_–Ap–ALN when compared to SiO_2_–Ap particles. These changes can be explained considering that the alkyl chains with amino groups attached present in the ALN structure are surface-exposed in the resulted SiO_2_–Ap–ALN particles.

Moreover, TG analysis performed in the inert atmosphere also revealed the differences in the profiles of both materials. Up to 200 °C, SiO_2_–Ap as well as SiO_2_–Ap–ALN particles lost about 9% of their initial mass (see Figure 2C). As can be noticed, the TG profile for SiO_2_–Ap shows much slower decomposition and desorption of the organics at temperatures above 250 °C. These results might suggest that the organic groups of ALN molecules bound to SiO_2_–Ap–ALN particles are exposed to the material’s surface. These findings correspond well to XPS measurements, confirming the presence of organic groups on the surface. The total mass losses (%) calculated based on the obtained TGA results in the inert atmosphere are 19.2 and 22.5% for the SiO_2_–Ap and SiO_2_–Ap–ALN particles, respectively. Thus, the weight content of anchored ALN estimated based on the increase of the final weight loss in TG profiles was about 3.3% (see Figure 2C). This value was lower than that obtained using spectrophotometric determination of the ALN content in the synthesized particles amounting to about 6%.

In our previous paper, we have performed the TG analysis for silica particles and observed two distinct mass loss steps in the obtained thermogram; the first one mainly connected with the removal of the physically adsorbed water from the silica surface (up to 130 °C) and the second one reflecting a slow condensation of silanols (above 190 °C).^26^ The estimated total mass loss was equal to 11%. Herein, we have used amine-functionalized silica particles with propyl groups exposed on the surface, thus the total weight loss for SiO_2_–Ap was higher. The TG profile for HAP presented by Ma et al. revealed only 1% weight loss. Therefore, the main weight changes in the particles developed by us originated from functionalized silica and the presence of ALN. Comparing the XRD pattern for SiO_2_–Ap and SiO_2_–Ap–ALN particles, no noticeable changes could be observed (Figure 2B). These results are in line with previous findings that conjugation of ALN had little influence on the crystal structure of Ap.^27^ There was no variation observed in the microstructures of particles after ALN conjugation using the SEM technique (Figure 2A). All data presented above confirmed the successful conjugation of ALN to bioactive silica–Ap particles under the experimental conditions employed.

### 3.2 Hybrid Materials Fabrication and Physicochemical Characterization

#### 3.2.1 Swelling in PBS

**Figure 3:**
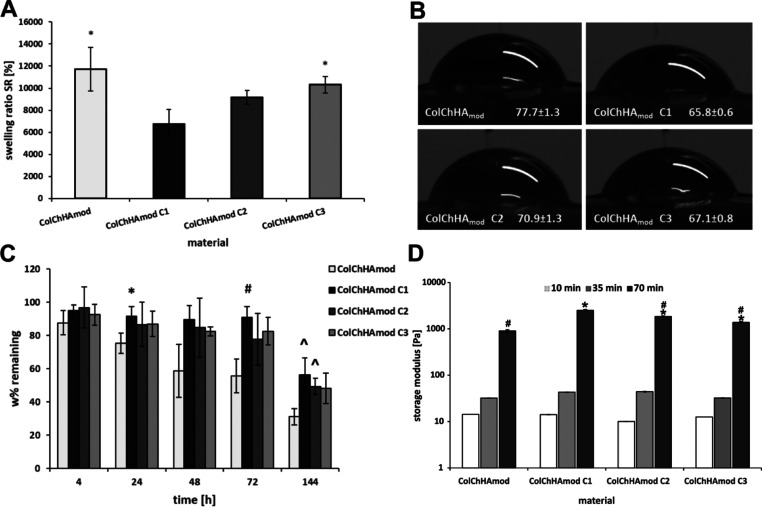
(A) SR for the pristine hydrogel and hybrid materials with the sodium ALN carrier incubated in PBS for 24 h. * indicates statistical significance when compared with ColChHA_mod_ C1. (B) Contact angle values (deg) measured on surfaces of tested materials. (C) Changes in the pristine hydrogel and hybrid material weight during 144 h of enzymatic degradation. Statistical analysis was carried out by comparing all types of materials at the same time of degradation. * indicates statistical significance when compared with ColChHA_mod_ after 24 h of experiment, # indicates statistical significance when compared with ColChHA_mod_ after 72 h of experiment, and ^ indicates statistical significance when compared with ColChHA_mod_ after 144 h of experiment. (D) Values of the storage modulus (*G*′) measured in 10, 35, and 70 min after starting the experiment are presented on a logarithmic scale. Statistical significance was calculated using Student’s *t*-test. A comparison between two means was analyzed with a statistical significance level set at *p* < 0.05; * indicates statistical significance when compared with ColChHA_mod_ after 70 min and # indicates statistical significance when compared with ColChHA_mod_ after 70 min.

Swelling ability is one of the essential properties of hydrogel-based materials that has an impact on their use in both tissue engineering and drug delivery.^28,29^ In this study, swelling behavior of the developed materials was tested in PBS, and the calculated swelling ratios (SR) are shown in Figure 3A. The ColChHA_mod_ hydrogel with a Col/Ch/HAmod weight ratio equal to 50/20/30 was characterized by a very high degree of swelling. The result is affected by the polymer composition used, especially by the content of HAmod, as demonstrated in the previous work.^30^ Incorporation of the silica particles–Ap–sodium ALN system into a hydrogel structure reduces the ability of that material to swell. In particular, the SR for the hybrid material with the highest sodium ALN carrier concentration (ColChHA_mod_ C1) is significantly lower compared to the SR for the ColChHA_mod_ hydrogel. The relatively small amount of ALN in the whole hybrid materials should not affect the swelling properties, Jiang et al. showed that this drug had not relevant influence on the SR in tested hydrogels.^31^ In our case, the main factor causing the decrease in swelling of hybrid materials seems to be the silica particle–Ap system. We have shown in the earlier work that the swelling capacity of the hydrogels decreased with the increase in the silica particle content.^15^ Similar results were obtained by Zareie et al.^32^ that Ap may also contribute to limiting water adsorption. The addition of HAp in the form of nanocrystals as well as the larger aggregates resulted also in lowering the swelling of materials based on polysaccharides and their derivatives, such as alginate and carboxymethylcellulose.^33,34^ In the case of hybrid materials obtained in this study, the combination of silica particles and Ap forms (an inorganic solid phase) may limit the mobility of polymer chains and reduce free volumes in the polymer network. These in turn may result in lower water uptake. It should be noted, however, that the hybrid materials obtained are still able to swell to a considerable extent due to the presence of the Col/Ch/HAmod-based matrix.

#### 3.2.2 Wettability

Wettability of biomaterials is a significant feature of their surfaces affecting the adsorption of proteins and preservation of their conformation allowing for a positive cell response.^35^ Contact angle measurements were carried out to evaluate the wettability of the tested materials (Figure 3B). The average value of the contact angle for the hydrogel ColChHA_mod_ was 77.7°, while for hybrid materials (ColChHA_mod_ C1–C3) it was in the range of 65.8–70.9°. The addition of the SiO_2_–Ap–ALN system to the hydrogel matrix caused a decrease in the contact angle values, although all samples are still characterized by moderate wettability. The increased surface hydrophilicity of hybrid materials compared to the pristine hydrogel can be due to the hydrophilic nature of the individual components of the sodium ALN carrier. Lower contact angle values and therefore enhanced hydrophilicity are observed for materials containing silica,^15^ HAp,^36^ as well as sodium ALN.^37^ Maintaining hydrophilicity is important because hydrophilic surfaces bind fibronectin, which is responsible for attaching cells to the ECM. According to the literature, contact angles in the range of about 60–70° appear to be the most optimal for cell adhesion.^38^

#### 3.2.3 Enzymatic Degradation

Enzymatic degradation of the obtained materials was carried out using a collagenase solution, monitoring the weight loss for 144 h (Figure 3C). The weight of pristine hydrogel (ColChHA_mod_) decreases gradually at each time point of the test. After 144 h of immersion in collagenase solution, the remaining weight of the hydrogel was about 31% of the initial mass. The kinetics of the degradation process was different for hybrid materials (ColChHA_mod_ C1–C3). During the first 72 h of the study, no significant weight loss was observed, the remaining weight was in the range of 96–77%. Greater weight losses were noted, however, at the latter stage of the study. After 144 h, the remaining weight was in the range of about 56–48% of the initial weight. It should be noted that the degree of degradation for hybrid materials does not depend on the concentration of the SiO_2_–Ap–ALN system, because no significant differences in weight loss were observed between the hybrid materials at individual time points of the study. Addition of the sodium ALN carrier slowed the degradation process of hybrid materials during treatment with collagenase solution compared to the pristine hydrogel, the largest differences were observed after 72 and 144 h. These results are consistent with the swelling properties described earlier. The SiO_2_–Ap–ALN system increases the stability of the hydrogel materials, a more compact structure with smaller free volumes inside the structures not only causes lower water uptake, but also makes it difficult for collagenase to reach the most sensitive hydrogel components.^39^ As a result, the enzymatic degradation for hybrid materials (ColChHA_mod_ C1–C3) is slower compared to that for the ColChHA_mod_ hydrogel.

#### 3.2.4 Microstructural Characterization (SEM)

SEM images of the material surfaces are shown in Figure S3. The ColChHA_mod_ hydrogel without the addition of the sodium ALN carrier is characterized by well-exposed polymeric fibers and a porous structure. The microstructure of hybrid materials (ColChHAmod C1–C3) appears to be more compact, and their polymer networks are definitely more tightened compared to the pristine hydrogel one. The presence of the SiO_2_–Ap–ALN system is clearly visible for all hybrid materials obtained, even for ColChHAmod C3 with the lowest carrier concentration. The SiO_2_–Ap–ALN systems occur in the form of smaller and larger aggregates with spherical shapes, integrated into the whole polymeric network. The SEM images confirm that the microstructure of hybrid materials may indeed affect the degree of swelling and enzymatic degradation as suggested above.

#### 3.2.5 Rheological Evaluation

The materials developed in this work were designed to serve as an injectable hydrogel-based hybrid system for the combined application of osteoporosis treatment and bone regeneration. Our concept is based on the hypothesis that the hybrid materials in the form of viscous sols can be introduced in a minimally invasive way (by injection) to the diseased tissue, where they will be in situ gelated under physiological conditions, thus forming a scaffold for cell growth while simultaneously supplying locally the necessary drug. In order to verify that hypothesis, we have performed the rheological measurements for hybrid materials, and by observing the changes in the storage modulus (*G*′) with time, we were able to confirm the transition from the sol to the gel state. The values of the storage modulus measured at 10, 35, and 70 min after starting the experiment are presented in Figure 3D. As shown at the beginning of the gelation process (10 min after preparation of the mixture), the elastic modulus values for all composites are at a low level (in the range of 10–14 Pa) confirming their viscoelastic state and the injectable form. The *G*′ values increase significantly after 35 min, reaching a maximum value within 70 min after starting the cross-linking process (gel formation). Thus, a comparison of the *G*′ values at the beginning and at the end of the rheological experiment proves that the developed materials can serve as injectable systems, which is especially important when considering their future TE applications.

Importantly, the obtained results reveal that addition of bioactive SiO_2_–Ap–ALN particles into a biopolymeric matrix substantially improves the mechanical properties of materials developed. Statistically significant differences were found for the *G*′ value after 70 min of gelation for all hybrid materials compared to the elasticity module (*G*′ after 70 min) obtained for the control material (ColChHA_mod,_*G*′ = 900 Pa) (see Figure 3D). The uppermost storage modulus determined after 70 min of gelation was observed for the system with the highest SiO_2_–Ap–ALN content (ColChHA_mod_ C1, *G*′ = 2500 Pa) (statistical significance when compared with the *G*′ after 70 min for all studied materials). It should be emphasized that in our previous work on the hydrogels structurally amended via covalent attachment of surface-modified silica particles, we have not observed such a tendency.^15^ Our findings were that silica particles during the cross-linking process created some aggregates affecting the organization of the matrix network causing the decrease of the elastic modulus values of the final hybrids. That observation was in line with the Alvarez et al.^40^ results. They revealed that silica particles at a higher concentration form aggregates that induced perturbation in the Col fibrillary organization. Consequently, the elastic properties of the resulted silica nanoparticle–Col composites deteriorated. Herein, we have demonstrated that SiO_2_–Ap–ALN particles, in the studied concentration range, do not provoke the appearance of such heterogeneities in the hybrid network that could be the failure points under mechanical stress and deteriorate the materials’ elastic properties. Furthermore, the XPS results confirmed the presence of amine groups surface-exposed on the SiO_2_–Ap–ALN particles that can take part in the cross-linking process. Therefore, such an increase in the storage modulus for hybrids may also indicate efficient cross-linking between the bioactive ALN carriers and the biopolymeric matrix upon genipin treatment. Overall, our findings reveal that by adjusting the SiO_2_–Ap–ALN content, the mechanical properties of the multifunctional organic–inorganic hybrids might be adjusted to the defined needs.

### 3.3 Drug Release Studies

**Figure 4:**
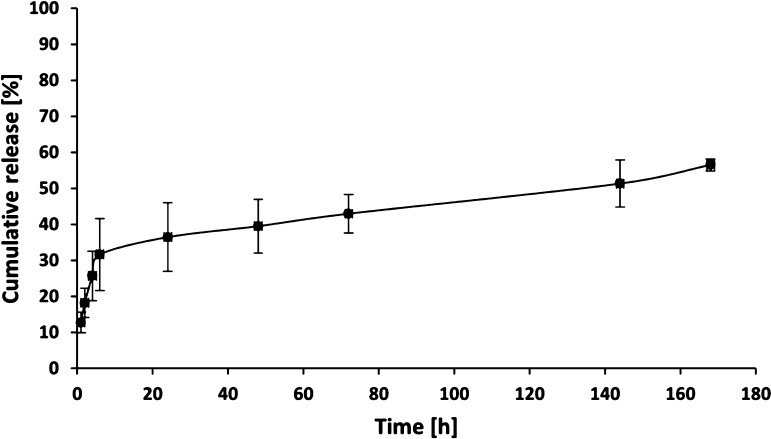
Cumulative release of ALN from hybrid material ColChHA_mod_ C1.

Materials developed in this work were designed to serve as a drug delivery system; the ALN release from a hybrid with the highest concentration of SiO_2_–Ap–ALN (ColChHA_mod_ C1) was also evaluated. The cumulative release profile in PBS is presented in Figure 4. The study showed a burst release at initial time points, followed by a slower prolonged release. The average initial burst amounted approximately to 31% of the cumulative release after 6 h of testing. That result may be due to the fact that in the obtained materials, the SiO_2_–Ap–ALN nanoparticles are also present in their surface layers, as indicated by SEM imaging and wettability studies. That facilitates the process of ALN release from these structures observed at the early stages of the experiment. Nevertheless, the relatively slow degradation of the matrix and the compact structure of the hybrid material may contribute to the prolonged release from the inner part of the material in the later stages of the experiment. The average cumulative release of ALN is approximately 56% after 168 h of testing. These results demonstrated that the placement of the SiO_2_–Ap–ALN system in the hydrogel matrix enhances functions of the scaffold. The hybrid materials enable the prolongation of ALN release, potentially supporting gradual tissue regeneration with a therapeutic effect. To determine the release mechanism, three commonly applied kinetic models for drug release were used—Higuchi, Ritger–Peppas, and Weibull models. Their mathematical description and the obtained parameter values are shown in Table 1. Higuchi and Ritger–Peppas models are short-time approximations and were fitted to 60% of the release data.^41,42^ A better fit was demonstrated for the Ritger–Peppas model due to the higher *R*^2^ value. In this model, *n* is an exponent characterizing various release mechanisms. For *n* ≤ 0.45, the mechanism of release is consistent with the kinetics of Fickian diffusion, the range 0.45 < *n* > 0.89 means anomalous (non-Fickian) diffusion, and for *n* = 0.89, the drug release mechanism was case II transport.^43^ The parameter *n* determined for our system was 0.33, which indicates the Fickian diffusion mechanism of the drug release in the developed hybrid material. In the next step, the Weibull model was used to fit the entire release data, in which *b* is the shape parameter indicating the release process from the polymer matrix. If *b* < 0.75, the dominant mechanism is Fickian diffusion, while for 0.75 < *b* < 1 a diffusion is combined with swelling-control transport.^39^ In our system, *b* was 0.30, which indicates Fickian diffusion as the primary release mechanism and this result is in agreement with that obtained using the Ritger–Peppas model.

**Table 1:** Parameters for the Kinetic Models Used^a^

mathematical model	parameters	
Higuchi 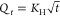	*K*_H_	5.63
	*R*^2^	0.7944
Ritger–Peppas *Q*_*t*_ = *K*_RP_*t*^*n*^	*K*_RP_	14.54
	*N*	0.33
	*R*^2^	0.8885
Weibull *Q*_*t*_ = 1 – exp(−*t*^*b*^/*a*)	*A*	5.89
	*B*	0.30
	*R*^2^	0.9418

a *Q*_*t*_—the amount of drug released in time *t*, *K*_H_, *K*_RP_—the release rate constants, *n*—the release exponent, *a*—the scale parameter, *b*—the shape parameter, and *R*^2^—the coefficient of determination.

### 3.4 Biomineralization Study Employing the SBF Model

**Figure 5:**
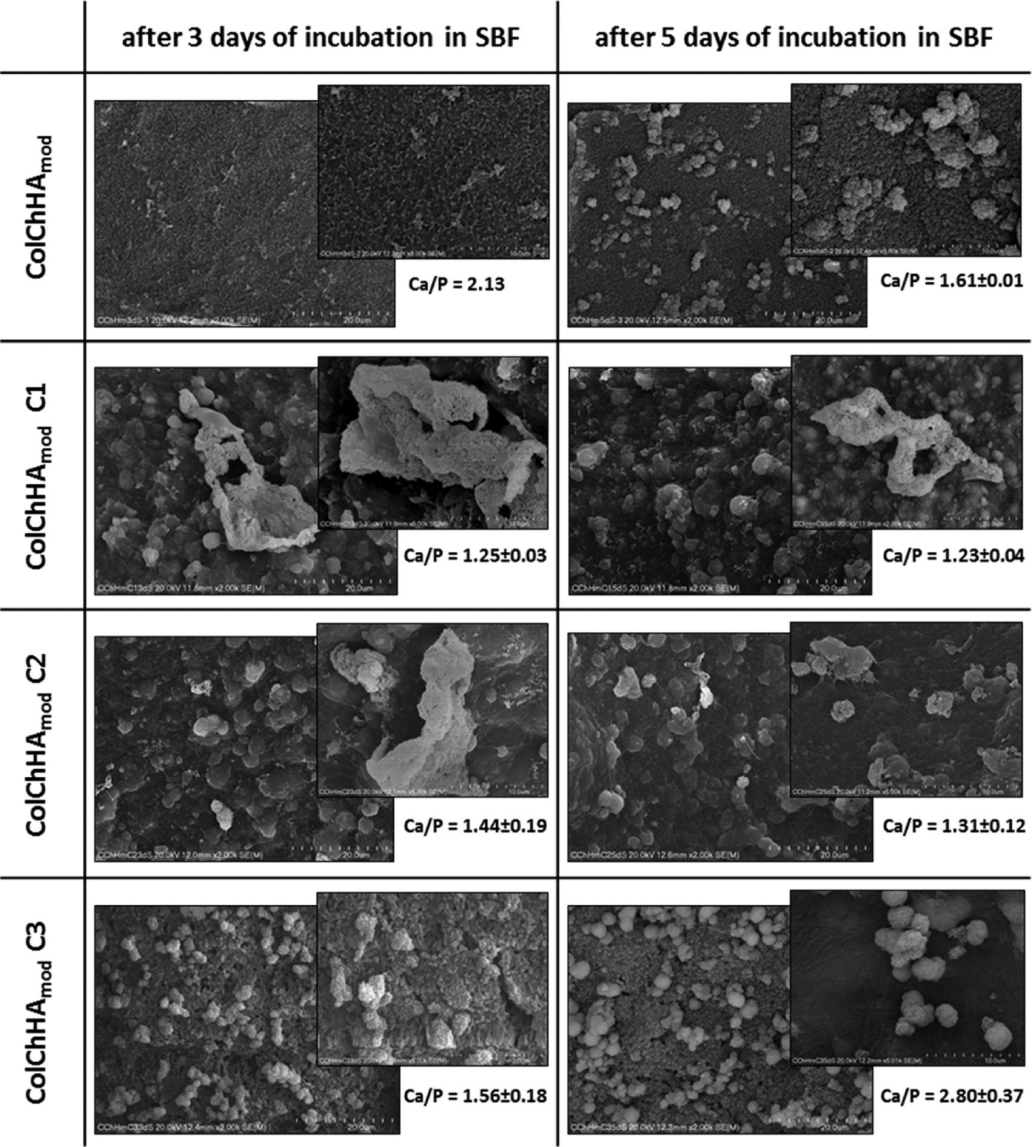
SEM images of the surface of pristine hydrogel and hybrid materials with the sodium ALN carrier after 3 and 5 days of incubation in SBF. The Ca/P ratios determined by EDS analyses.

Scaffolds for bone tissue regeneration should be bioactive. The bioactivity of the materials developed here can be determined by investigating the biomineralization process, which involves the deposition of an Ap layer on the surface of scaffolds immersed in a SBF solution.^44^ The building block of bone is calcium phosphate, in particular HAp. The stoichiometric HAp is characterized by the Ca/P ratio equal to 1.67, while the mineral phase in natural bones has the Ca/P ratio in the range of 1.5–1.7.^45^ For effective Ap formation, scaffolds should provide functional groups that can induce nucleation of the new mineral phase.^46^ Biomineralization studies of the obtained materials were carried out by their incubation in simulated plasma (SBF). Figure 5 shows the SEM images after 3 and 5 days of incubation in SBF and the Ca/P ratios determined by EDS analyses. The appearance of new mineral phases can be observed on all types of tested materials. In the case of pristine hydrogel (ColChHA_mod_), the initial nucleation of the new mineral phase is visible over the entire surface after 3 days of incubation. The high Ca/P ratio (2.13) indicates excess calcium in the formed phase. After 5 days of incubation, cauliflower-like structures were observed, as the individual objects or as the larger clusters, on the surface of the hydrogel. The Ca/P ratio for these structures was about 1.61, which is definitely smaller than the Ca/P ratio after 3 days and simultaneously very similar to that of stoichiometric HAp. Hyaluronic acid seems to be the main factor affecting enhanced biomineralization in that sample. It is known that the carboxyl groups (−COOH) present in the structure of hyaluronic acid can serve as the ion-binding sites, which lead to nucleation and to an increase in calcium phosphate on the surface of the hydrogel.^47^ The content of hyaluronic acid in the composition of the hydrogel and its surface exposure are of great importance because no mineral phase formation was observed in the case of hydrogels containing a much lower content of hyaluronic acid even after 7 days of incubation in SBF.^15^ The surface of the hybrid material with the lowest addition of the sodium ALN carrier (ColChHA_mod_ C3) looks similar to the pristine hydrogel, except that the process of forming cauliflower structures is faster (after 3 days of incubation). Interestingly, for this sample, there is also a large change in the Ca/P ratio between days 3 and 5 (from 1.56 to 2.80). The very high Ca/P ratio after 5 days of incubation differs significantly from the ratios of typical calcium phosphates. A large prevalence of calcium over phosphorus may result from the coexistence of CaO formation together with another mineral phase.^48^ In the case of hybrid materials with a higher SiO_2_–Ap–ALN concentration (ColChHA_mod_ C1 and ColChHA_mod_ C2), a different mineralization process can be observed. New mineral phases appeared after 3 days of incubating these materials in SBF, and they were rather in the form of larger individual structures with irregular shapes. The Ca/P ratios after 3 and 5 days of incubation are in the ranges 1.25–1.23 and 1.44–1.31 for ColChHA_mod_ C1 and ColChHA_mod_ C2, respectively. They are close to the ratios in compounds such as octacalcium phosphate (OCP, Ca/P ratio of 1.33) and tricalcium phosphate (Ca/P ratio of 1.5).^43^ It should be emphasized that bioactivity of ColChHA_mod_ C1 and ColChHA_mod_ C2 containing the SiO_2_–Ap–ALN system is much higher than that of previously studied hybrid materials containing only silica particles.^15^ For those materials, the mineral phase with a Ca/P ratio of 1.29 formed only after 7 days of incubation in SBF. In the current study, a similar mineral phase is already observed at the surfaces of materials after 3 days of incubation. Enhanced bioactivity is the result of the presence of the SiO_2_–Ap–ALN system in the hydrogel network, in particular the content of Ap. HAp is characterized by osteoconductive properties and provides a nucleation site for further formation of the Ap structure, therefore it is widely used as an inorganic component of composites with improved biomineralization for bone tissue engineering applications.^49,50^ Sodium ALN, which has high affinity for bone minerals, can be an additional factor favorably affecting biomineralization.^51^ To sum up this aspect of our studies, one can conclude that the addition of the sodium ALN carrier to the hydrogel matrix improves the bioactivity of the obtained materials while maintaining the therapeutic properties of ALN.

### 3.5 In Vitro Biological Evaluation of Developed Hybrids

**Figure 6:**
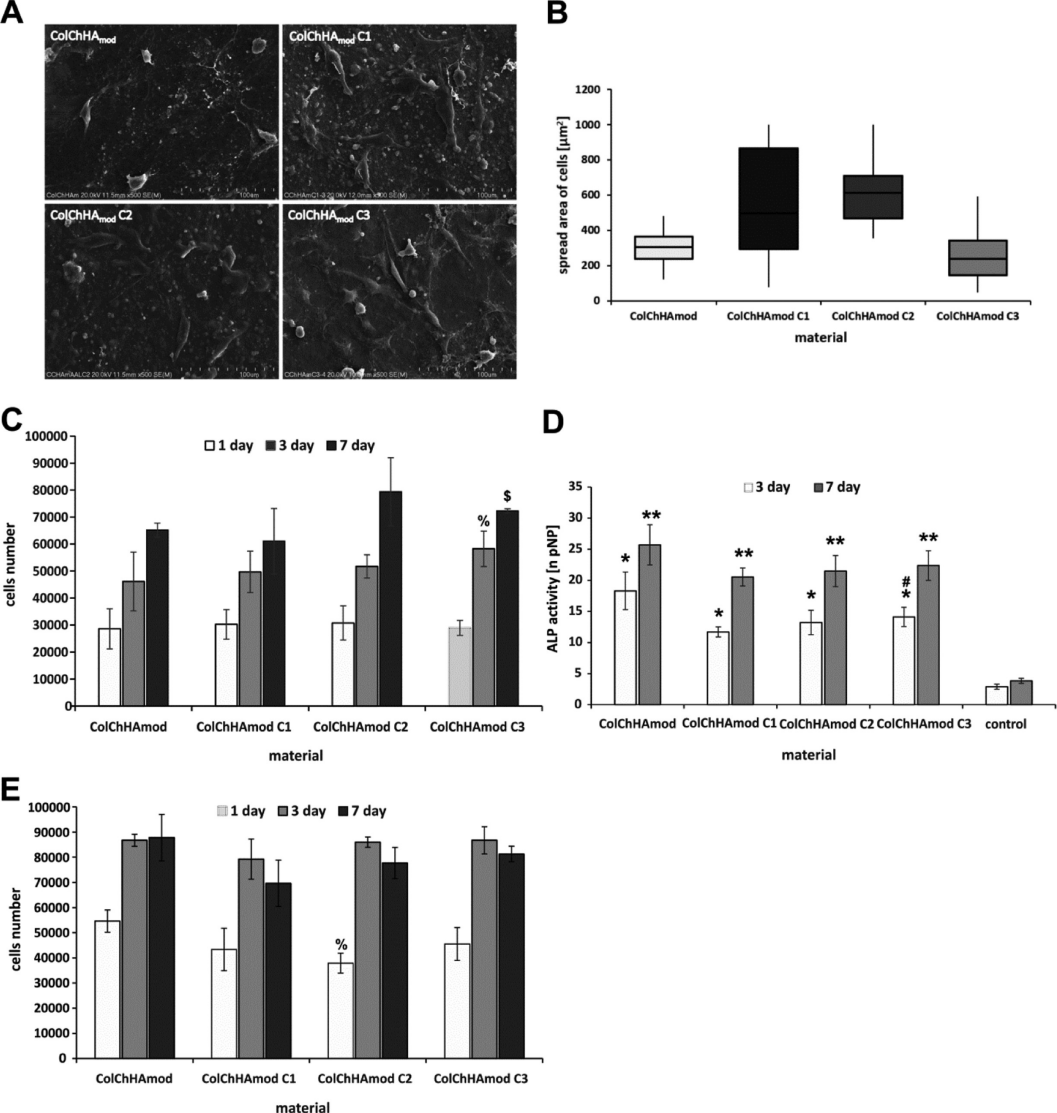
(A) SEM images of the MG-63 cell morphology on the surface of the pristine hydrogel and hybrid materials with the sodium ALN carrier. (B) Distributions of the spread area for MG-63 cells cultured on the pristine hydrogel and hybrid materials with the sodium ALN carrier. The area of cells was calculated employing ImageJ software. Boxes represent interquartile range and median. (C) Cell number and (D) ALP activity of MG-63 cells grown on the surface of the materials studied on days 1, 3, and 7 of the culturing. Statistical significance was calculated using Student’s *t*-test. In (C), % indicates statistical significance when compared with ColChHA_mod_ on day 3 and $ indicates statistical significance when compared with ColChHA_mod_ on day 7. In (D), * indicates statistical significance when compared with control on day 3, ** indicates statistical significance when compared with control on day 7, and # indicates statistical significance when compared with ColChHA_mod_ C1 on day 3. Cells cultured on the tissue culture plate were considered as control. (E) Cell number of J774A.1 cells grown on the surface of the materials studied on days 1, 3, and 7 of culturing. Statistical significance was calculated using Student’s *t*-test. % indicates statistical significance when compared with ColChHA_mod_ on day 1.

One of the expected biological roles of developed materials in bone tissue regeneration is the inhibition of the bone resorption phase by impairing the osteoclast function while simultaneously maintaining bone formation by supporting osteoblast proliferation on its substrate. Therefore, we have performed preliminary studies on the biocompatibility of prepared hybrids utilizing MG-63 cells as a model. This cell line is very often used in studies focusing on the characterization of materials fabricated for potential bone tissue engineering applications.^12^ Interactions between cells and scaffolds are among the key elements of research in the field of tissue engineering. Surface properties such as topography, material stiffness, or surface functional groups affect cell adhesion and morphology. Cell adhesion is an essential step that allows further cell growth, proliferation, and differentiation.^52^ SEM images show the morphology of MG-63 cells cultured on the surface of the ColChHA_mod_ hydrogel and on the ColChHA_mod_ C1–C3 hybrid materials (Figure 6A). The degree of cell flattening varies for the individual materials—the cells adopt spherical or more elongated and flattened shapes. In particular, it can be observed that on ColChHA_mod_ and ColChHA_mod_ C3, the cells are less flattened and occupy smaller areas compared to those on hybrid materials with higher sodium ALN carrier concentrations (ColChHA_mod_ C1 and ColChHA_mod_ C2). Determination of the cell spread area distributions for all tested materials confirmed the above observations (Figure 6B). In the case of ColChHA_mod_ C1 and ColChHA_mod_ C2, the average spread area of cell is higher than those for ColChHA_mod_ and ColChHA_mod_ C3. In addition, the hybrid material with the largest content of the sodium ALN carrier (ColChHA_mod_ C1) is characterized by the largest variation in the cell spread area, whereas the cells on the pristine hydrogel (ColChHA_mod_) and material with the lowest carrier concentration (ColChHA_mod_ C3) have a relatively narrow distribution of the spread area. Differences in the degree of cell adhesion and their morphology may be related to the content of the SiO_2_–Ap–ALN system. In our previous work, we showed that silica particles incorporated into the hydrogel matrix do not have a significant effect on the shape and spread area of MG-63 cells.^15^ Therefore, the Ap seems to have the greatest impact on differences in the cell morphology on the obtained hybrid materials. It is well known that combining natural polymers with HAp is a promising approach in scaffold design for bone tissue engineering.^53^ HAp exposed on the surface of such systems supports the integration of cells with the scaffold, in particular, it is demonstrated that this component plays an essential role in the initial stages of cellular activity, that is, adhesion and proliferation.^25^ It is possible that sodium ALN also contributes to greater cell spreading on the surface of hybrid materials due to the positive effect on osteoblast activity if it is used at a low dose.^54^

We have quantitatively assessed the ability of developed materials to support MG-63 cell line proliferation. The results of viability tests performed in a biological experiment employing the AB assay at 1st, 3rd, and 7th culture days are depicted in Figure 6C. We found that the presence of SiO_2_–Ap–ALN particles at the concentration tested does not deteriorate the biocompatibility of hybrids with respect to the control ColChHA_mod_. A similar variation tendency with the prolongation of the experimental time for MG-63 cells cultured on all types of materials prepared was revealed. The number of viable cells substantially grew up after 3 days of culturing and reached the maximum at day 7. Moreover, a comparable increase in the number of cells (no statistically significant differences) was observed in the following days of the experiment (1, 3, and 7) on hybrid materials with C1 and C2 concentrations of the SiO_2_–Ap–ALN particles when compared to the control (ColChHAmod). In order to gain better insights into the functions of MG-63 cells cultured on the tested materials, we have additionally studied the alkaline phosphate (ALP) activity that serves as one of the markers confirming the osteoblastic phenotype and mineralization. The results of ALP activity measurements performed on days 3 and 7 are illustrated in Figure 6D. For all analyzed materials, the level of ALP increased after 7 days of culturing. It was also observed that ALP activity for cells cultured on tested materials was significantly higher compared to that for cells on the tissue culture plate at both time points used for observation (on days 3 and 7) (statistical significance). We have found a similar tendency in our previous work.^15^ Moreover, analyzing the impact of the SiO_2_–Ap–ALN concentration on ALP activity, statistically significant differences were revealed only for the ColChHAmod C3 material on day 3 (when compared to ColChHAmod C1 day 3). There were no significant differences in ALP activity between the hybrid systems after 7 days of the biological experiment.

Overall, our findings clearly demonstrated that SiO_2_–Ap–AN particles at the concentration studied do not decrease the biocompatibility of the ColChHA_mod_-based hybrids. We showed that obtained materials support the proliferation of MG-63 cultured on their surfaces and ALP expression. We noticed that the addition of SiO_2_–Ap–ALN particles to the ColChHA_mod_ matrix does not enhance ALP activity compared to the pure hydrogel. Park et al. revealed that ALN has the potential to stimulate an early and late osteogenic differentiation.^55^ They showed that ALP activity of MG-63 cells grown on biphasic calcium phosphate (BCP) scaffolds containing ALN was significantly higher than those grown on pure BCP. Moreover, it has also been established that some inorganic particles (HAp, silica) can influence the kinetics and conformation of protein adsorbed from the culture medium and thus improve the cell metabolic activities.^13,14^ We have not observed the noticeable impact of the ALN concentration nor the content of SiO_2_–Ap–ALN particles on the ALP activity secreted by the cells cultured on the hybrid materials. This could be related to the presence of HA in hybrids developed and its affinity to osteoblasts.^56^ HA regulates their cellular responses including proliferation, differentiation, and adhesion by interacting with cell surface receptors and with binding proteins.^57^ Taking into account the above-presented data, one may conclude that in experiments that lasted for 7 days, the HA played a key role in MG-63 differentiation into the cells expressing the osteoblastic phenotype and in regulating the ALP activity secreted by these cells.

#### 3.5.1 Biological Evaluation of Hybrids in Osteoclast-like Cell Culture In Vitro

In order to demonstrate the ability of the developed hybrid materials for inhibition of bone resorption, the preliminary in vitro study employing model osteoclast-like cells, J774A.1, was performed. Because the main features of J774A.1 cells are similar to those of osteoclast, they act as a reference cell line for the detailed analysis of BP metabolism.^10,58^ The results of the viability test (AB) carried out on the 1st, 3rd, and 7th days of cell culturing on the surface of materials are depicted in Figure 6E. The preliminary biological evaluation reveals the proliferation inhibition of osteoclast-like cells with respect to the control sample (pristine ColChHA_mod_) after 7 days of experiment. Moreover, it was noticed that this tendency is dependent on the SiO_2_–Ap–ALN particle concentration and is most pronounced for the ColChHA_mod_ C1 hybrid. Thus, it was demonstrated that hybrids with SiO_2_–Ap–ALN particles at the concentration studied (C1, C2, and C3) exhibit therapeutic potential revealed by the inhibition of J774A.1 activity. Our findings also confirmed that ALN is able to effect the formation of bone cells while being introduced as SiO_2_–Ap–ALN particles dispersed in hydrogels. Hence, taking into account the type of developed formulations, it will be possible to induce the local drug action, thus increasing the efficacy of therapy while reducing the drawbacks of systemic action following the oral delivery of ALN.

### 3.6 Biological Evaluation In Vivo

Considering the potential future applications of the developed materials, it seems necessary to perform biological evaluation in vivo. Based on the results of physicochemical characterization as well as in vitro biological studies, the hybrid with the highest SiO_2_–Ap–ALN concentration (ColChHAmod C1) and the pristine ColChHAmod hydrogel as a control was selected for further biological research. The experiments on the mouse model were performed to evaluate the biocompatibility of the selected systems and examine the potential and safety of the obtained materials under in vivo conditions. The injectability as well as the ability to gel in vivo were verified, while the panel of biochemical and histopathological analyses enabled the determination of hemo-, hepato-, or nephrotoxicity of the developed systems.

#### 3.6.1 Hydrogel-Based Hybrid Materials Are Injectable and Are Sustainably Degraded In Vivo

**Figure 7:**
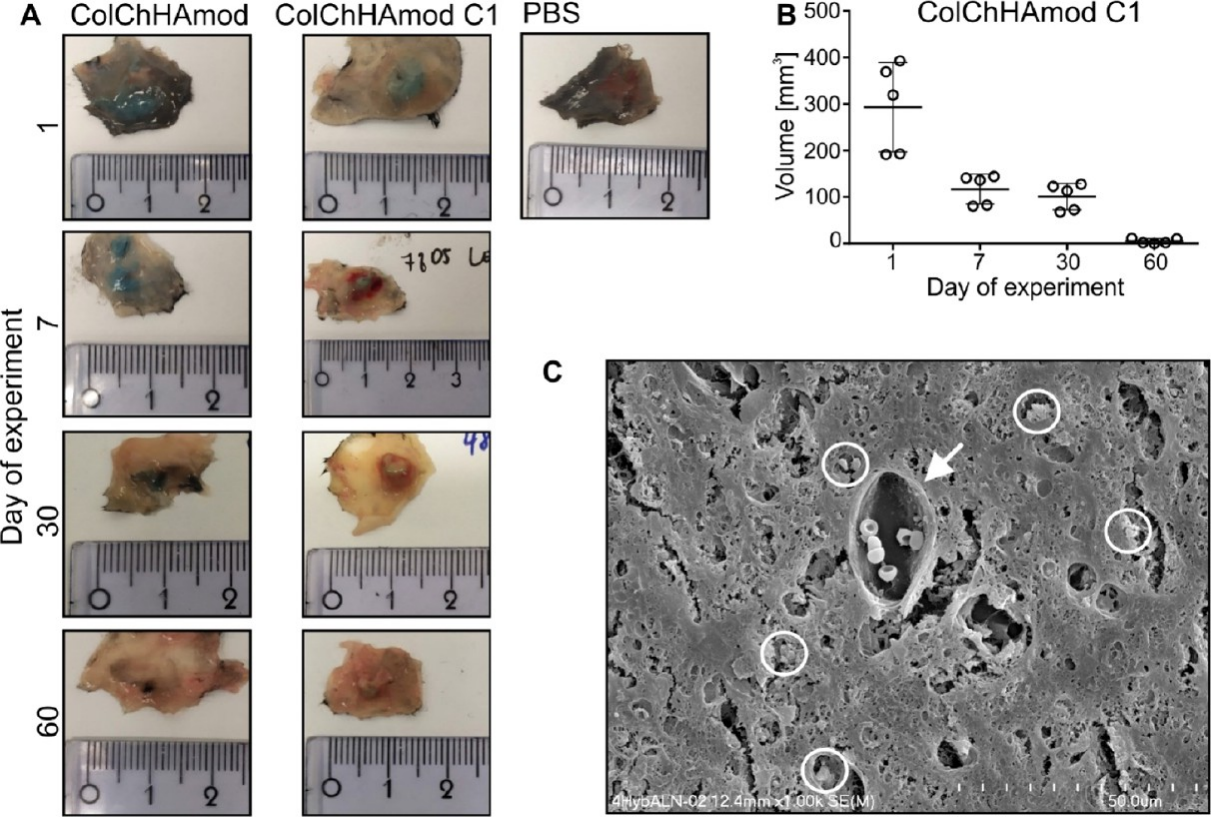
(A) Fragments of isolated skin with hydrogel-based materials. Animals were subcutaneously injected with PBS or with hydrogels and euthanized after various periods. Skin fragments containing hydrogels were isolated, photographed, and then processed for further analyses. Representative pictures taken after various periods show changes in the hydrogels’ size, structure, and color, and indicate hydrogel degradation (*n* = 10). (B) Graph shows a decrease in the hydrogel volume observed after various periods followed by material injection. Each point on the graph represents an individual animal; each group’s line represents mean ± SD (*n* = 5). (C) Representative picture of ColChHAmodC1 isolated from mice skin after 60 days postinjection taken using a scanning electron microscope. The white circle indicates HAp aggregates as determined by EDS analysis. The white arrow points out the blood vessel in the hydrogel.

In in vivo studies, the tested materials were injected subcutaneously (right flank, shoulder area) into the healthy C57Bl/6 mice. Before administration, all components were mixed, transferred into a syringe, and incubated for 15 min at 37 °C (to induce gel formation). After incubation, the color of the tested materials changed from light gray to blue-green; all the materials remained in the liquid phase, hence no problems were encountered with their subcutaneous administration. Moreover, no hydrogel leakage was observed immediately after administration (through the hole created when the needle was removed), and the entire mixture was injected. It was, therefore, confirmed that all the tested materials had very good injectability. Twenty-four hours after the materials were given to the animals, their presence at the injection site was confirmed. First, the structure of hydrogels was examined by the palpation method. Due to the dense composition of ColChHAmod C1, it could be detected through the animal’s skin. On the other hand, ColChHAmod had a tissue-like structure; thus, it was imperceptible during palpation. Mice were sacrificed at 24 h, and 7, 30, and 60 days after material injection (1st day of the experiment, 7th, 30th, 60th days of the experiment), and both ColChHAmod and ColChHAmod C1 were visualized after skin removal (Figure 7A). The color of both tested materials was blue. ColChHAmod was softer, more hydrated, and had a desirably less-compact structure than ColChHAmod C1. We observed the progress of volume change quantitively over time only for ColChHAmod C1 (it was dense enough for palpation examination and measurements with a caliper). A significant, rapid decrease in the ColChHAmod C1 volume was observed after 7 days (volume was 2.5 times smaller than that observed after 1 day postinjection), indicating its biodegradation (Figure 7B). Between 7 and 30 days of the experiment, the volume of ColChHAmod C1 did not change significantly, and finally, at the end of the experiment lasting 60 days, the volume was almost 60 times smaller than that observed after 1 day postinjection (Figure 7B). Also, ColChHAmod C1 had no gel consistency anymore, and this material was hard, light blue-green in color, and blood vessels were observed near it. We also confirmed significant postmortem degradation of ColChHAmod. On the 60th day of the experiment, the material no longer resembled a gel; its debris/residues were visible under the skin as black fibers. Additionally, we examined the structure isolated from skin ColChHAmod and ColChHAmod C1 by SEM. The results show a loose arrangement of the fibers in the ColChHAmod and dense composition of ColChHAmodC1.

The staining of tissue sections containing the ColChHAmod C1 hydrogel with Alizarin red made it possible to visualize the calcium deposits present in HAp, a component of this hydrogel (Figure S4). We observed that HAp was present in the samples collected at each observation point (1, 7, 30, and 60 days) of the experiment. Because tissue regeneration is a long process, this observation provides essential information about the long-term presence of HAp (osteogenic and osteoinductive substances) after hydrogel subcutaneous injection. This observation was also confirmed using SEM (see Figures 7C and S4). For sections obtained after ColChHAmod administration, no characteristic dark orange foci were observed after Alizarin red staining.

#### 3.6.2 ColChHAmod and ColChHAmod C1 Do Not Cause Systemic Toxicity

**Figure 8:**
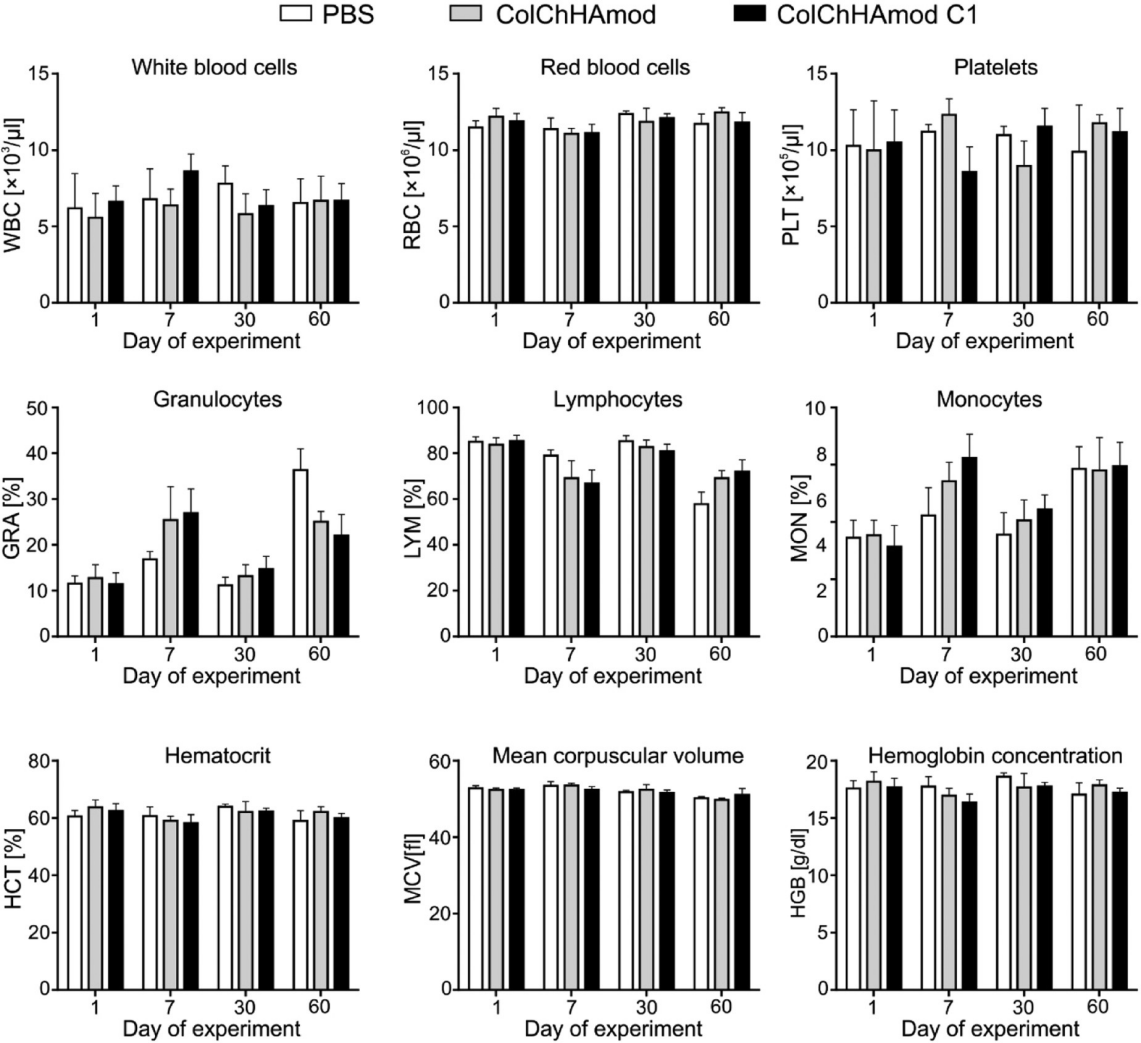
Blood hematology analyses performed for mice exposed to hydrogels. Animals injected with PBS or with materials studied were euthanized after various periods. Blood was taken before euthanasia from the facial vein. Bars for each group represent the mean ± SD (*n* = 7).

**Figure 9:**
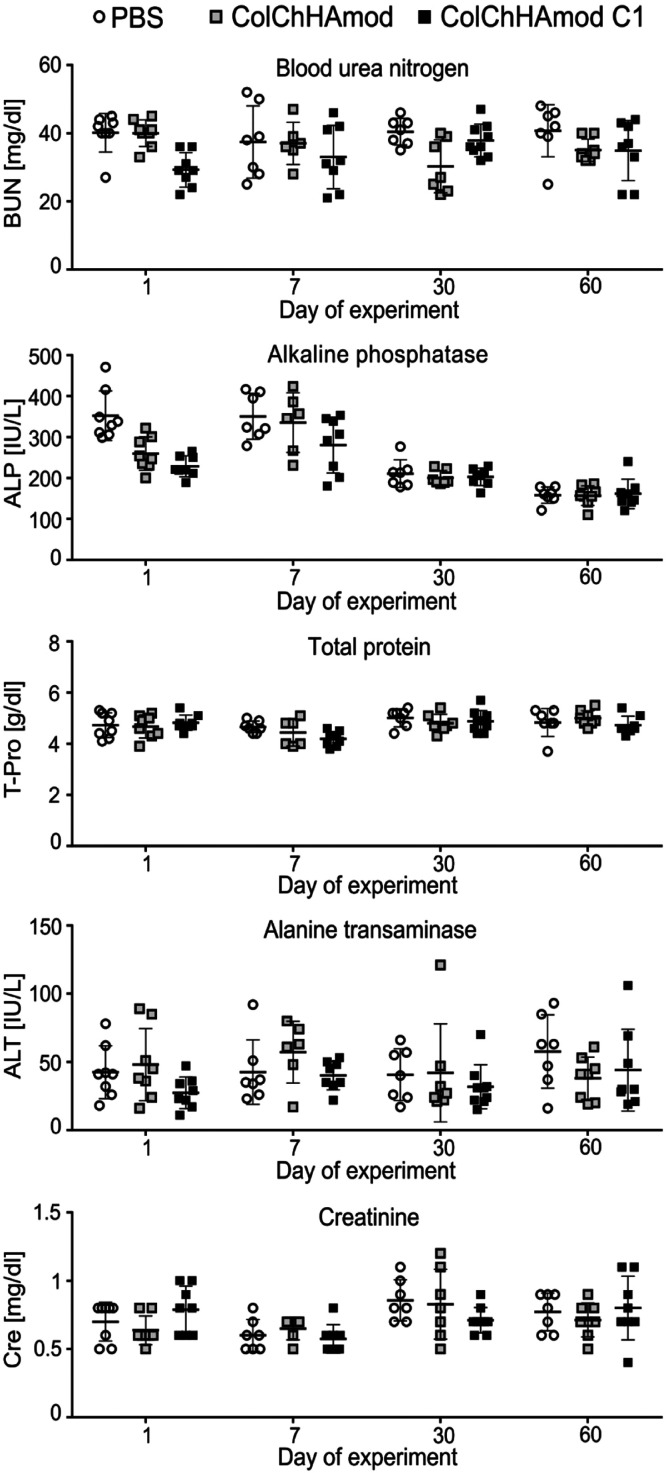
Biochemical serum analyses performed for mice exposed to hydrogels. Animals were subcutaneously injected with PBS or with materials studied and euthanized after various periods. Blood was taken after euthanasia by cardiac puncture, and sera were isolated. Each point on the graph represents an individual animal; each group’s line represents mean ± SD (*n* = 7).

Analysis of systemic biocompatibility aimed to exclude adverse reactions provoked by subcutaneous administration of materials and products of their degradation. The biopolymers are expected degradation products in the case of ColChHAmod, while in the case of ColChHAmod C1, additionally released products could be HAp, ALN, or silica released from the SiO_2_–Ap–ALN particles. Although the materials have been injected subcutaneously, their degradation products may cause systemic toxicity by entering the bloodstream. The animals were euthanized at different times after administering the materials (1, 7, 30, and 60 days), which allowed investigating the potential acute and chronic toxicity. No weight loss or disturbing changes in the animals’ appearance and behavior were observed during the experiment [data not shown]. As shown in Figures 8 and 9, no changes were observed in the blood morphology or in the activity or concentration of hepatotoxicity (ALT, T-Pro), nephrotoxicity (BUN, Cre), or other tissue damage (ALP) markers.

Moreover, the histopathological examination excluded changes in the morphology of the selected organs isolated after 30 or 60 day posthydrogel administration (Figures S5 and S6).

**Figure 10:**
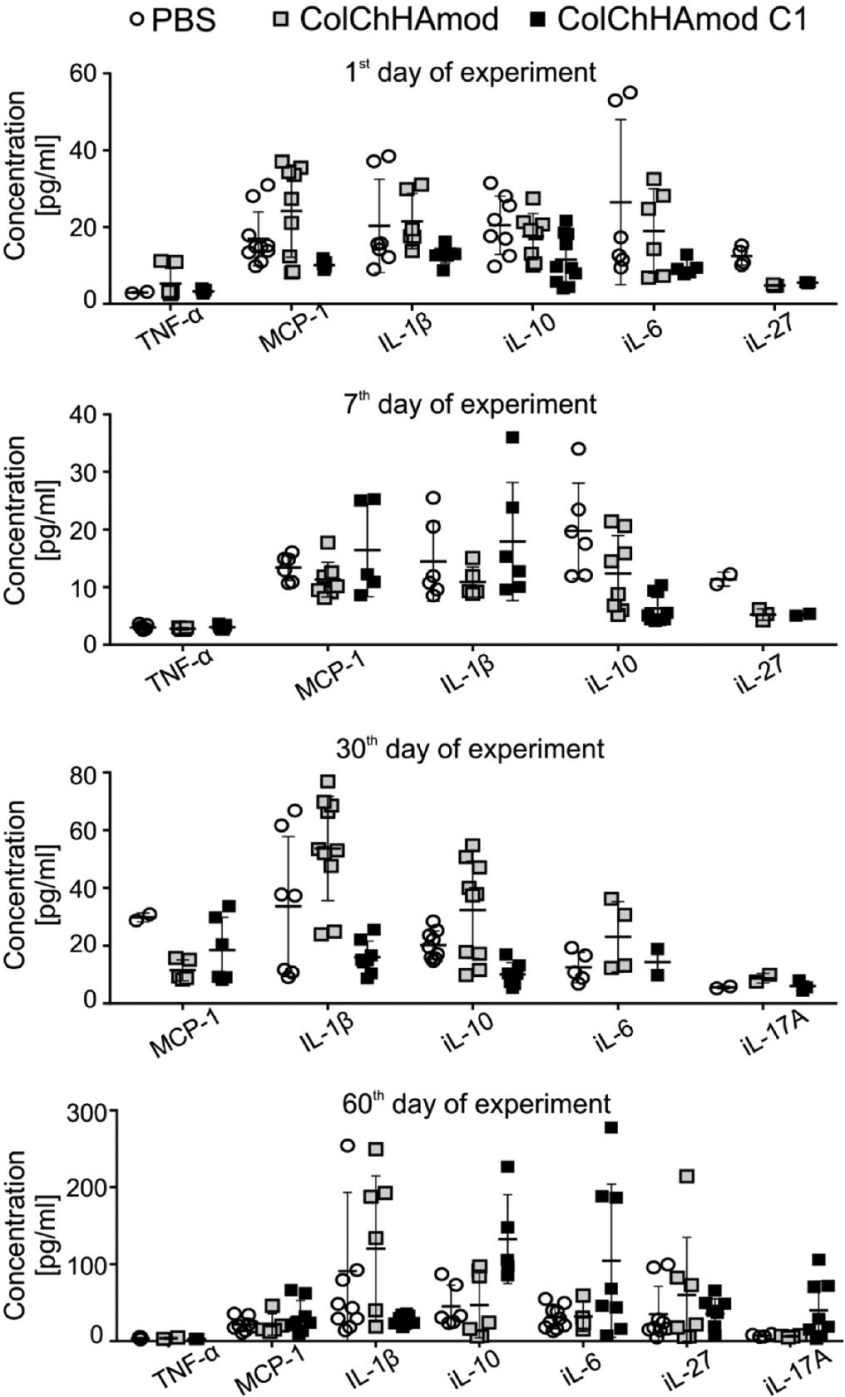
Cytokine detection in sera after materials administration. Animals were subcutaneously injected with PBS or with hydrogels and euthanized after various periods. Blood was taken after euthanasia by cardiac puncture, and sera were isolated. Each point on the graph represents an individual animal; each group’s line represents mean ± SD. Only values that were within the detection range were plot in the graph. For some cytokines, the indicated concentration was below the detection level (but all were analyzed according to the LEGENDplex 13-plex Mouse inflammatory panel). Analyzed *n* = 10, but the graph has a notable different value of *n* = 2–10.

Finally, the serum concentrations of cytokines, including proinflammatory cytokines, confirmed the absence of immunotoxicity of subcutaneously administered hydrogels or products of their degradation (Figure 10). Our results indicate a lack of systemic toxicity that could result from the administration of hydrogel containing potentially toxic products of degradation to animals. They, therefore, confirm that the use of biomaterials loaded with SiO_2_–Ap–ALN can be a promising method of repair of osteoporotic bone without the risk of systemic toxicity caused by the drugs or other products of material degradation. Our data correspond well to the results of other studies in which the ALN immobilized on a graphene oxide-functionalized Col sponge, Ch/β-glycerophosphate, or tetra-PEG hydrogels was administered to the experimental animals.^5,59^

#### 3.6.3 Biological Changes Occurring within the Hydrogels and Their Interaction with Cells In Vivo

**Figure 11:**
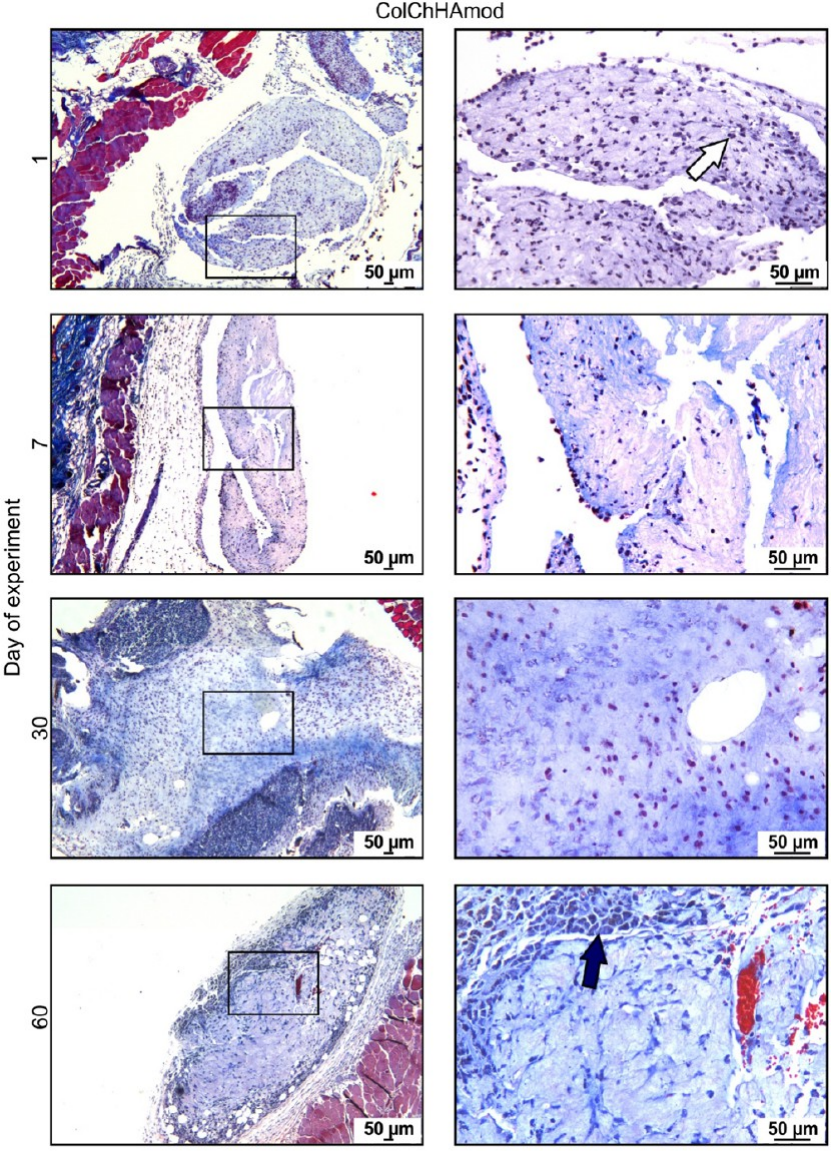
Hydrogel ColChHAmod stained with Masson’s trichrome. Animals were subcutaneously injected with PBS or with materials and euthanized after various periods. Skin fragments containing hydrogels were isolated, processed, and stained with Masson’s trichrome. The white arrow indicates neutrophils and dark blue arrow indicates macrophages.

**Figure 12:**
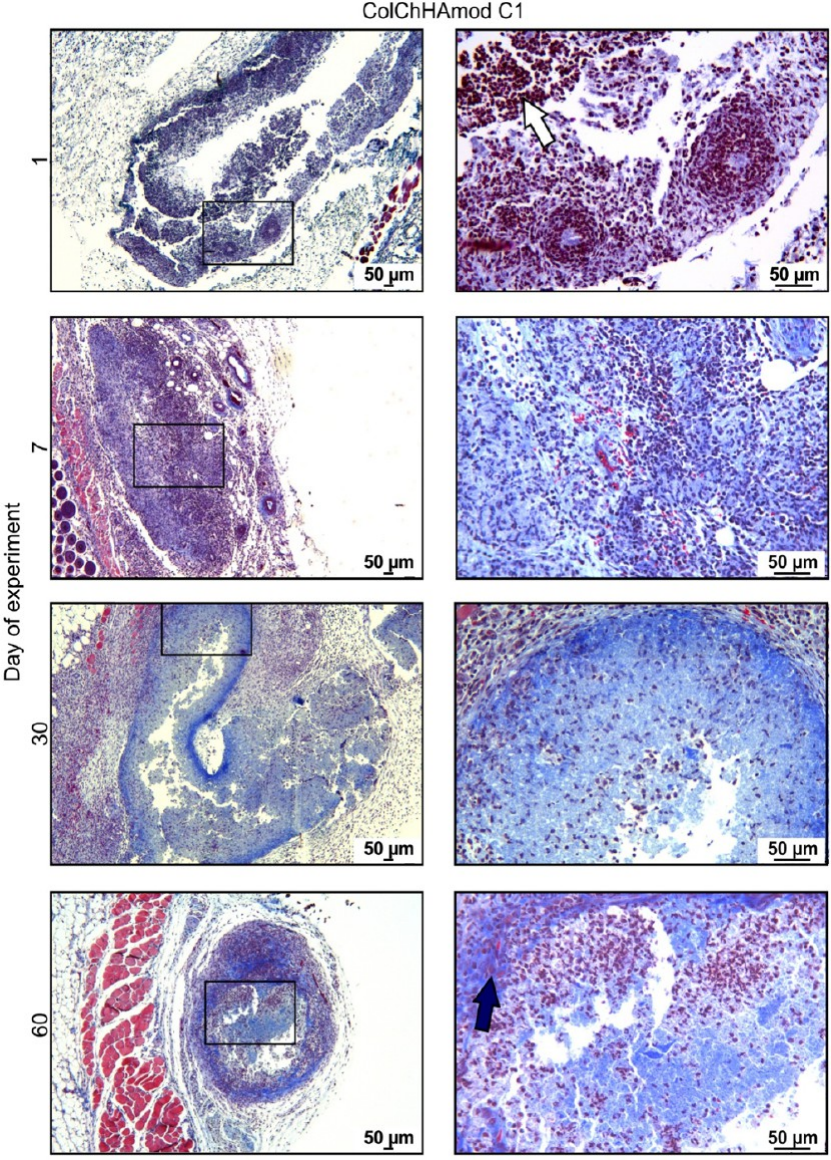
Hydrogel ColChHAmod C1 stained with Masson’s trichrome. Animals were subcutaneously injected with PBS or with materials and euthanized after various periods. Skin fragments containing hydrogels were isolated, processed, and stained with Masson’s trichrome to visualize hydrogel infiltration by host cells and detect Col. The square represents an area that is shown at a larger magnification. The white arrow indicates neutrophils and the dark blue arrow indicates macrophages.

We then investigated the changes in the tested materials (isolated with skin fragments) at various times after their administration. First of all, we paid attention to the recruitment of the host cells to the materials. Twenty-four hours after the subcutaneous administration of ColChHAmod and ColChHAmod C1, we observed an influx of immune cells (mainly neutrophils) responsible for developing local inflammation (Figures 11 and 12). Immune cells were present in materials at all time points. However, a longer time after the biomaterials’ administration, more leukocyte populations were recruited (leading to the resolution of inflammation). Therefore, on the 7th day after administration, macrophages and LYM were also visible in addition to neutrophils. The influx of immune cells occurs throughout the material volume; however, this phenomenon was much more intense at the periphery of materials (the deeper, the fewer cells are visible). With ColChHAmod C1, a more intense inflammation was observed. The result was the production by fibroblasts of animal skin a clear layer of Col (fibrotic capsule formation) around the hydrogel with SiO_2_–Ap–ALN, visible on the 30th and 60th days after administering the material. This phenomenon was much less severe with the ColChHAmod hydrogel. For the ColChHAmod material, inhibition of the proinflammatory response was observed on the 60th day after administration. After the 30th day of the experiment, leukocytoclasia was observed, indicating the death of immune cells at the site of inflammation. We also observed newly created blood vessels in the isolated sections of materials 60 days after the administration. The presence of blood vessels within the materials was also confirmed by SEM analysis (see Figure 7C).

Because ColChHAmod C1 contains complex SiO_2_–HAp–ALN particles, it is difficult to identify which component is responsible for inflammation. There are a lot of reports underlying the role of HAp in the immune response. For example, the results indicating induction of local inflammation have been observed after subcutaneously injecting the HAp nanoparticles. At the site of administration of these nanoparticles, increased recruitment of immune cells resulting in fibrotic capsules formation was confirmed.^60^ Velard et al., studying the proinflammatory activity of HAp nanoparticles, showed that they are an enormously stimulating factor of polymorphonuclear neutrophils. After implantation of biomaterials containing HAp, the quick activation and recruitment of neutrophils were observed, which correspond first to cytokine secretion (IL-1a and IL-8) and then appearance of chemokine (MIP-1a and MIP-1b) and intracellular matrix metalloprotease (e.g., MMP-9). That promotes the recruitment of subsequent subpopulations of leukocytes leading to HAp implant-associated inflammation.^61^ There are also reports on the proinflammatory activity of silica, which is linked with the induction of reactive oxygen species either extra- or intracellularly (due to the reactive silanol groups and surface-associated radicals) and subsequent activation of the proinflammatory response.^62^ Finally, ALN is a multifunctional drug with osteoclast inhibiting as well as immune cell stimulation properties.^63^

Overall, we did not observe the systemic proinflammatory response manifested by elevated proinflammatory cytokines in the blood (as it is demonstrated in Figure 10). Thus, our results indicate that only local inflammation can be induced after administering the tested materials.

## 4 Conclusions

The increasing demand for multifunctional materials with defined physicochemical and biological features for TE is very often in conflict with the complexity and high costs of fabrication. These unfortunately caused a strong gap between the research and the real applications. We believe that the hybrids developed herein represent a promising alternative to formulations investigated so far for the combined application of osteoporosis treatment and bone regeneration due to their low production cost, ease of implantation, and multifunctional biological functionality. The synthesized hybrid materials can be precisely and in a minimally invasive way introduced into the defect site (by injection), where they will create scaffolds enabling bone tissue reconstruction. Such designed hybrids chemically cross-linked with a compound of natural origin can serve as systems for the controlled, localized delivery of ALN that plays a key role in the treatment of osteoporosis. The developed formulation provides a non-invasive location of the ALN at the implantation site, while maintaining the structure, biological properties, and limiting the potential adverse effects of the oral therapy. We have demonstrated that hybrids obtained are efficient osteoconductive materials simultaneously possessing the ability for activation of tissue regeneration, represented by an in vitro model of osteoblast-like cells (MG-63), and inhibition of osteoclast-like cell proliferation (J7741.A). Moreover, the purposely selected biomimetic composition of these materials (biopolymer hydrogel enriched with the mineral phase) allows their biointegration (in vitro study using the SBF model) and ensures the desired physicochemical features (mechanical properties, wettability, and swellability). Finally, the biocompatibility studies revealed the potential and safety of developed materials under in vivo conditions. The results of these experiments indicated a lack of systemic toxicity of the developed systems and thus demonstrated that the use of a hybrid with SiO_2_–Ap–ALN can be a promising method for the repair of osteoporotic bone. Moreover, no systemic proinflammatory response which could be manifested by elevated proinflammatory cytokines in the blood after administering the tested materials was revealed. There was only local inflammation observed. The materials induced the mineralization process and, more importantly, angiogenesis—formation of new blood vessels—was observed after 60 days of experiment. It should be pointed out that the injectability of the materials studied, as well as the ability to gel in vivo, was also confirmed. Overall, it may be concluded that multifunctional hydrogel-based hybrid materials presented in this study and fabricated by a facile one-step procedure possess a set of qualities that render them promising candidates for the development of formulations for the combined application in osteoporosis treatment and bone regeneration.

## Supporting Information Available

Details of materials and methods; FTIR spectra of SiO_2_, SiO_2_–Ap, SiO_2_–Ap–ALN, and ALN; XPS wide spectra of SiO_2_–Ap and SiO_2_–Ap–ALN particles with high-resolution results corresponding to N 1s; XPS results showing atomic compositions of the SiO_2_–Ap and SiO_2_–Ap–ALN particles (in %); SEM images of microstructure of the pristine hydrogel and hybrid materials with sodium ALN carrier; images of HAp’s foci in tissue sections containing the ColChHAmod C1 hydrogel stained with Alizarin red; SEM images of hydrogel fragments isolated from the skin; and images presenting the histological examination of the liver, spleen, and kidney isolated from animals after 30 and 60 days postmaterial injection (PDF)
