# Supplementary material for: Addressing the Osteoporosis Problem—Multifunctional Injectable Hybrid Materials for Controlling Local Bone Tissue Remodeling
Source: ACS Appl Mater Interfaces. 2021 Oct 13;13(42):49762–79. doi: 10.1021/acsami.1c17472 (PMC8554765; doi:10.1021/acsami.1c17472)
Supplement: Supplementary file 1 — am1c17472_si_001.pdf [file am1c17472_si_001.pdf]

Supporting Information

Addressing the osteoporosis problem - multifunctional injectable hybrid materials for controlling the local bone tissue remodeling.

Adriana Gilarska[1,2], Alicja Hinz[3], Monika Bzowska[3], Grzegorz Dyduch[4], Kamil Kamiński[1], Maria Nowakowska[1], Joanna Lewandowska-Łańcucka[1]*

[1] Faculty of Chemistry, Jagiellonian University, Gronostajowa 2, 30-387 Kraków, Poland

[2] AGH University of Science and Technology, Faculty of Physics and Applied Computer Science, Mickiewicza 30, 30-059 Kraków, Poland

[3] Department of Cell Biochemistry, Faculty of Biochemistry, Biophysics and Biotechnology Jagiellonian University, Gronostajowa 7, 30-387 Kraków, Poland

[4] Department of Pathomorphology, Jagiellonian University Medical College, 30-387 Kraków, Poland

Corresponding author: lewandow@chemia.uj.edu.pl

2. Materials and methods

2.1. Materials

Collagen (**Col**) type I rat tail (3.5 mg/ml solution, BD Biosciences), chitosan (**Ch**) (low molecular weight, Sigma-Aldrich), hyaluronic acid (**HA**) (M_w ~ (1.5-1.8) x 10^6 Da, Sigma-Aldrich) was functionalized with lysine (**HA$_{mod}$**) according to the procedure described by us earlier[1] (to confirm the successful modification and to calculate the substitution degree (about 25%) elementary analysis, ^{1}HNMR and FTIR spectra were measured. Results of these experiments were presented in our previous work [1] (*3.1. Characterization of HA derivative (HAmod)* paragraph, Fig.1, Fig. S1 and Fig S2), genipin (Challenge Bioproducts Co., 98%), alendronate sodium trihydrate (4-amino-1-hydroxy-1-phosphonobutyl phosphonic acid, monosodium, Alendronate sodium trihydrate, **ALN**, Sigma Aldrich, pharmaceutical secondary standard), tetraethoxysilane (TEOS, ≥98%, Fluka), (3-aminopropyl)triethoxysilane (APS, 98%, Sigma Aldrich), acetic acid (Chempur), ethanol (99,8%, spectroscopic grade), sodium chloride, NaCl (POCh, p.a.), sodium hydrogen carbonate, $NaHCO_3$ (POCh, p.a.), potassium chloride, KCl (Chempur, p.a.), di-potassium hydrogen phosphate trihydrate, $K_2HPO_4 \cdot 3H_2O$ (Sigma Aldrich, 99%), magnesium chloride hexahydrate, $MgCl_2 \cdot 6H_2O$ (POCh,

p.a.), calcium chloride, $CaCl_2$ (Sigma Aldrich, 93%), sodium sulfate, Na_2SO4 (POCh, p.a.), tris-hydroxymethyl aminomethane, $((HOCH_2)_3CNH_2)$ (Tris) (Sigma Aldrich, 99,8%), hydrochloric acid 1M, HCl (POCh). Dulbecco's Modified Eagle Medium (DMEM, Sigma-Aldrich), penicillin-streptomycin solution (10.00 units/ml), fetal bovine serum (FBS, HyClone), trypsin (HyClone), Alamar Blue reagent (Invitrogen), Cell Digestion Buffer and Cell Assay Buffer compounds: tris-hydroxymethyl aminomethane, $((HOCH_2)_3CNH_2)$ (Tris) (Sigma Aldrich, 99.8%), zinc chloride, $ZnCl_2$ (POCh), magnesium chloride hexahydrate, $MgCl_2{\cdot}6H_2O$ (POCh), Triton X-100 (POCh)], p-nitrophenyl phosphate (pNPP) (Sigma-Aldrich), glutaraldehyde solution (Sigma-Aldrich), hexamethyldisilazane reagent grade (HMDS, Sigma-Aldrich), **osteoblasts-like cells: MG-63** (ATCC® CRL-1427™) (Organism: Homo sapiens; Tissue: bone; Disease: osteosarcoma) and **osteoclast-like cells: J774A.1** (ATCC® TIB-67™) (Organism: Mus musculus; Cell Type: macrophage; Disease: reticulum cell sarcoma) were from American Type Culture Collection. *Materials for in vivo biocompatibility studies:* Sigma-Aldrich provided formalin solution (10%, neutral, buffered); alcoholic solution of Eosin Y (with phloxine) and Alizarin Red S. Cytoseal XYL and Harris Hematoxylin Qpath were purchased from VWR Chemicals (Radnor, Pennsylvania, USA); Ethanol (EtOH) 96% and 99,8%, xylene, acetone, and other salts, acids and bases were from Avantor Performance Materials, Poland. Multiparameter strips: Spotchem II Panel V for sera biochemical analyses were from Woodley, and LEGENDplex Mouse Inflammation Panel (13-plex) immunoassay for cytokines detection was from Biolegend.

2.3. Methods

Silica–apatite (SiO_2-Ap) and silica-apatite-alendronate (SiO_2-Ap-ALN) particles characterization

The microstructure of the silica-based particles was evaluated by means of the cold field emission scanning electron microscope (**SEM**) HITACHI S-4700 equipped with a NORAN Vantage energy dispersion spectrometer (**EDS**). X-ray Photoelectron Spectroscopy (**XPS**) analysis was carried out using a multifunctional ESCA instrument equipped with additional accessories produced by PREVAC. Data analysis was performed using the CasaXPS program. The chemical composition of the developed materials surface was calculated from peak areas normalized on the basis of the acquisition parameters. X-ray diffraction (**XRD**) measurements was carried employing X'Pert PRO MPD diffractometer (PANalytical) with a Bragg–Brentano geometry. A copper x-ray sealed tube was used as the radiation source. Graphite

monochromator was applied to select only Cu Kα (1.540598 Å–Kα1 and Kα2–1.544426 Å) radiation. Thermogravimetry measurements (**TG**) and thermal decomposition of the materials were studied using a Mettler-Toledo TGA/SDTA851e thermogravimeter. The samples were heated at a rate of 10 ºC min^{-1} from zero to 1000 ºC in argon flow of 110 mL min^{-1}

Hybrid materials characterization

The swelling ability of the hydrogels was investigated under physiological conditions by materials incubation at 37°C in PBS buffer with gentle shaking for 24h. After that PBS buffer was removed, the hydrogels were rinsed twice with deionized water and weighed (Ws). Next, materials were dried by lyophilization and weighed again (Wd). The swelling ratio (SR) was calculated using the following equation: $SR = \frac{W_s - W_d}{W_d} \cdot 100\%$.

The **wettability** of obtained hydrogels was analyzed by contact angle measurements carried out using Surftens Universal instrument (OEG GmbH, Frankfurt, Germany). Five contact angle values were measured for each sample of hydrogels, and the average value was calculated. The **mechanical properties** of the tested hydrogels were studied with a Physica MCR-301 rheometer (Anton Paar) equipped with a parallel plate (Ø = 20 mm) made of stainless steel under conditions employed previously.[1] The measurements were carried out in oscillation mode utilizing a frequency of 1 Hz and a strain of 1%; the measuring gap was set at a distance of 1 mm. For **enzymatic degradation** evaluation, the materials studied were placed in 24-well plates and exposed to collagenase type I (0.2 mg/ml, 1ml, ≥125 U/mg) in 1X PBS with 0.36 mM $CaCl_2$ followed by incubation at 37°C and gentle shaking. At various time intervals, materials were weighted, and next, the fresh portion of enzyme solution was added to the system. For each sample, the experiments of degradation were carried out in triplicates, and the results are presented as the averages.

In vitro biomineralization

The prepared hybrids were transferred into 24-well plates, and 1 ml of freshly prepared SBF was added to each well. The plates were placed on a shaker table and incubated at 37°C for 3, 5, and 7 days (SBF was renewed every day). Next, SBF was removed, the materials were rinsed few times with deionized water and lyophilized for 24 hours. The microstructure of the hybrids developed after SBF treatment was evaluated by means of the scanning electron microscope (SEM) HITACHI S-4700 equipped with a NORAN Vantage energy dispersion

spectrometer. The obtained solid materials were stuck to the carbon tape on the silicone plate, and the thin film of gold was deposited on the sample by sputtering.

Biological experiments in vitro

Osteoblast-like (MG-63) and osteoclast-like (J774A.1) cells culture

MG-63 and J774A.1 cells were cultured in Dulbecco's Modified Eagle's Medium–high glucose with 4500 mg/L glucose, L-glutamine, sodium pyruvate, and sodium bicarbonate, liquid, sterile-filtered, suitable for cell culture (DMEM) supplemented with 10 % (v/v) fetal bovine serum (FBS) and 100 U/mL penicillin and 100 µg/mL streptomycin (HyClone) in a humidified atmosphere (90% humidity) with 5 % CO_2 at 37°C. Cells were cultured in the standard tissue culture flasks. The medium was changed three times weekly. Before seeding on the materials, MG-63 cells were washed twice with PBS solution and subsequently harvested after 3 min incubation with 1 mL of 0.25% trypsin with 0.1% EDTA. After adding 3 mL of DMEM (with 10% (v/v) FBS) the cell suspension was centrifuged at 1000 rpm for 5 min, the supernatant was removed, and the pellet was resuspended in the culture medium. For J774A.1 cells, subcultures were prepared by scraping. Next, the collected cell suspension was centrifuged and resuspended analogously as MG-63 cells.

Preparation of the material for cell culture

The series of developed materials (three samples for each type) were prepared in 24-well plate, washed twice with PBS and then sterilized using UV light for 20 min. Next, the wells were filled with medium (without serum) and left for about 1h in the incubator (37º C, 5% CO_2). Before cell culture, the medium was removed, and cells were seeded in the plate at a density of about $2x10^4$ cells per cm^2. The medium containing 90 vol% of DMEM (supplemented with 1 vol% of penicillin-streptomycin solution) and 10 vol% of serum was used.

Biological experiments in vivo

Animals used in the studies

Six-week-old C57BL/6 female mice were provided by the Animal Facility of the Faculty of Biochemistry, Biophysics and Biotechnology, Jagiellonian University, Kraków, Poland. Mice were housed under controlled conditions and provided with food and water ad libitum.

According to Polish law, all animal procedures were performed specifically to the Act on the Protection of Animals used for Scientific or Educational Purposes (D20150266L), which implements the European Parliament's Directive and the Council (2010/63/EU). All procedures agreed with the Institutional Animal Care and Use Committee (IACUC) guidelines and were approved by the 2nd Local IACUC in Kraków.

Figures and Table:

Figure S1. FTIR spectra of SiO_2, SiO_2-Ap, SiO_2-Ap-ALN and ALN respectively.

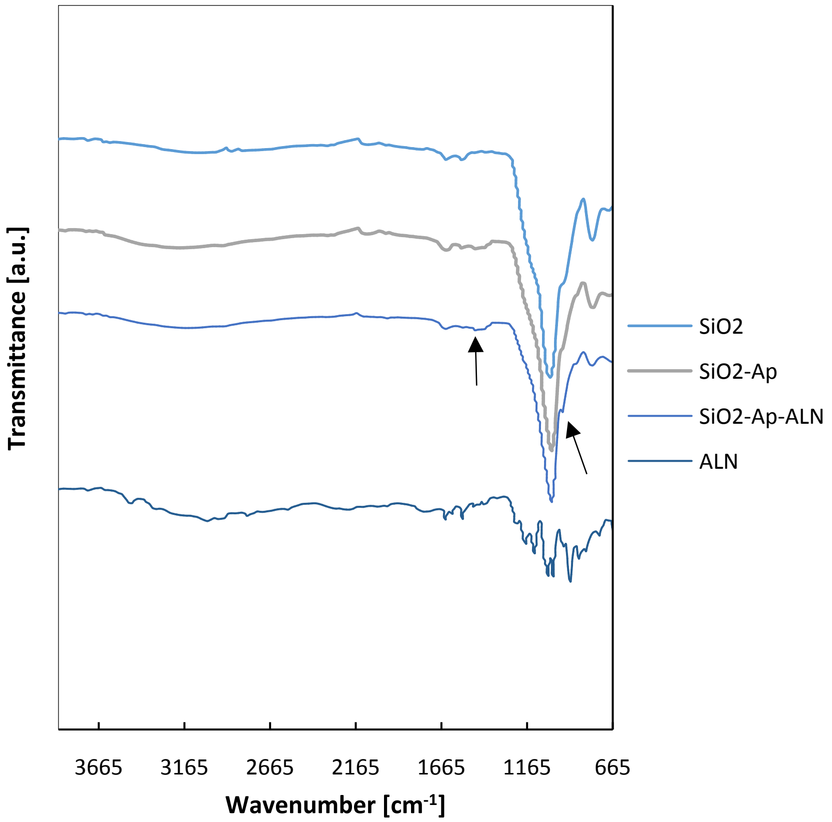

Figure S2. XPS wide spectra of SiO_2-Ap and SiO_2-Ap-ALN particles with high-resolution results corresponding to N 1s.

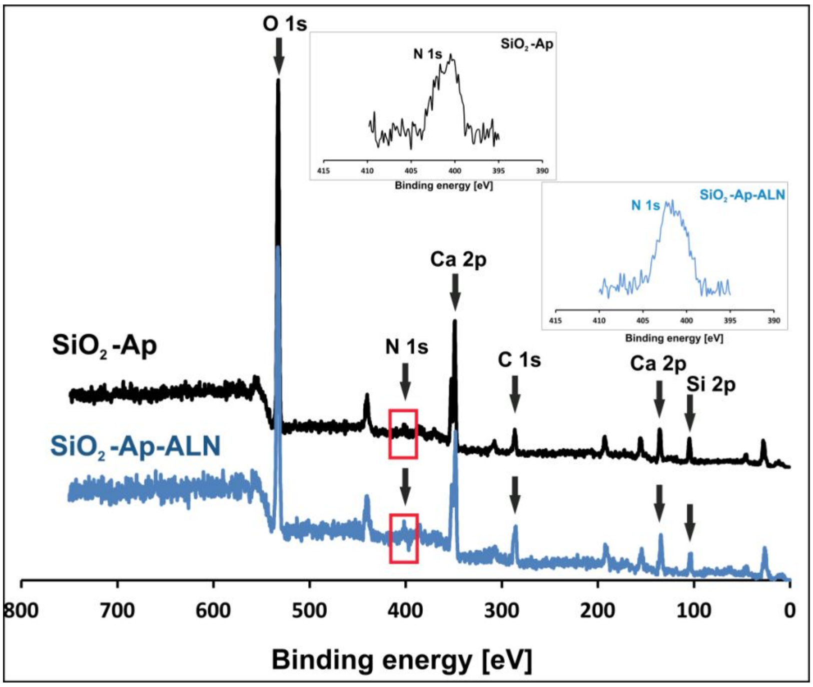

Table S1

XPS results showing atomic compositions of the SiO_2-Ap and SiO_2-Ap-ALN particles (in %).

**Atomic composition (%) of particles developed**	***O 1s***	***Si 2p***	***C 1s***	***N 1s***	***Ca 2p***	***P 2p***
SiO_2-Ap	62	3	4	1	26	4
SiO_2-Ap-ALN	60	3	8	2	23	4

Figure S3. SEM images of microstructure of the pristine hydrogel and hybrid materials with sodium alendronate carrier.

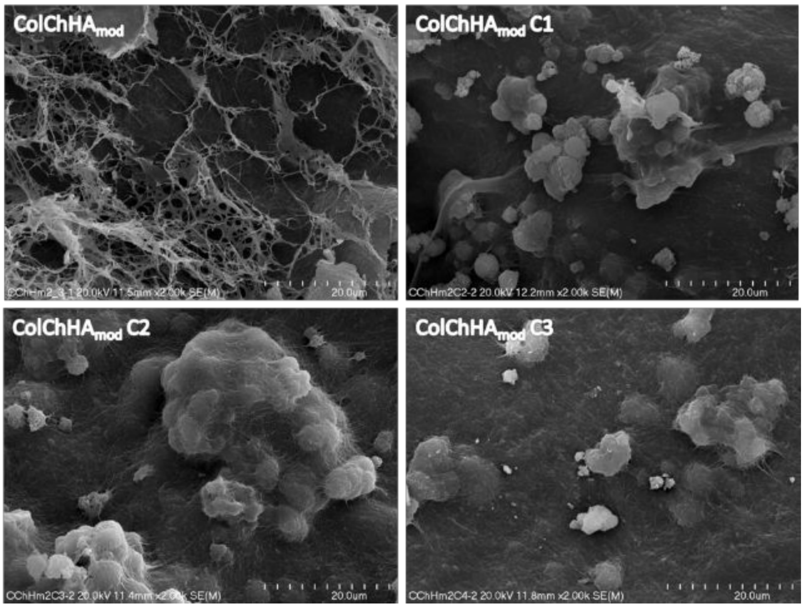

Figure S4. Visualization of hydroxyapatite's foci in tissue sections containing ColChHAmod C1 hydrogel. (A) Staining with Alizarin red. Animals were subcutaneously injected with PBS or with hydrogels and euthanized after various periods. Skin fragments containing hydrogels were isolated, processed, and stained with Alizarin red. Hydroxyapatite is visible in the hydrogel as dark orange foci. (B) Hydrogels' fragments isolated from skin and visualized by SEM.

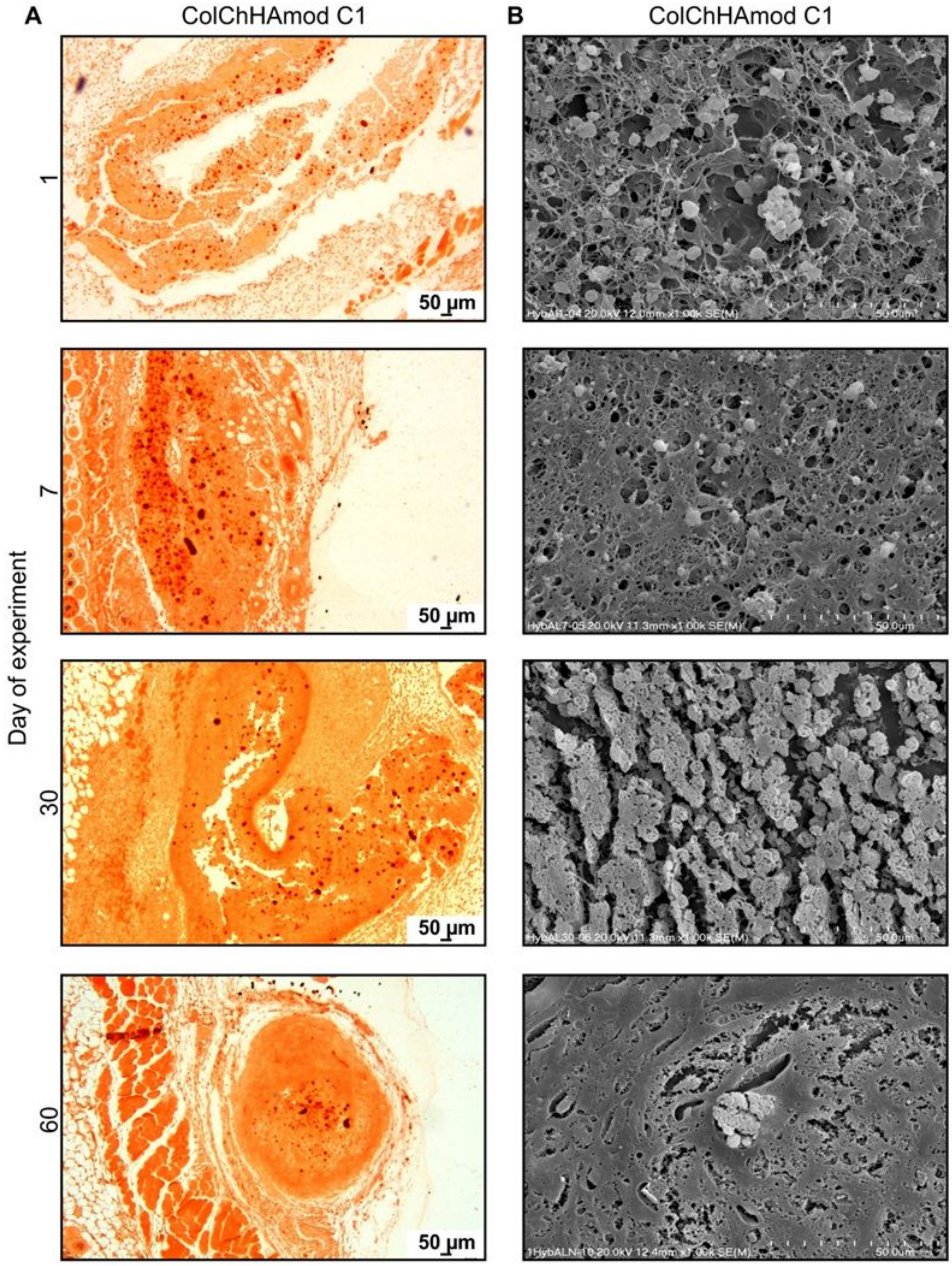

Figure S5. Histological examination of the liver, spleen, and kidney isolated from animals after 30 days post materials injection.

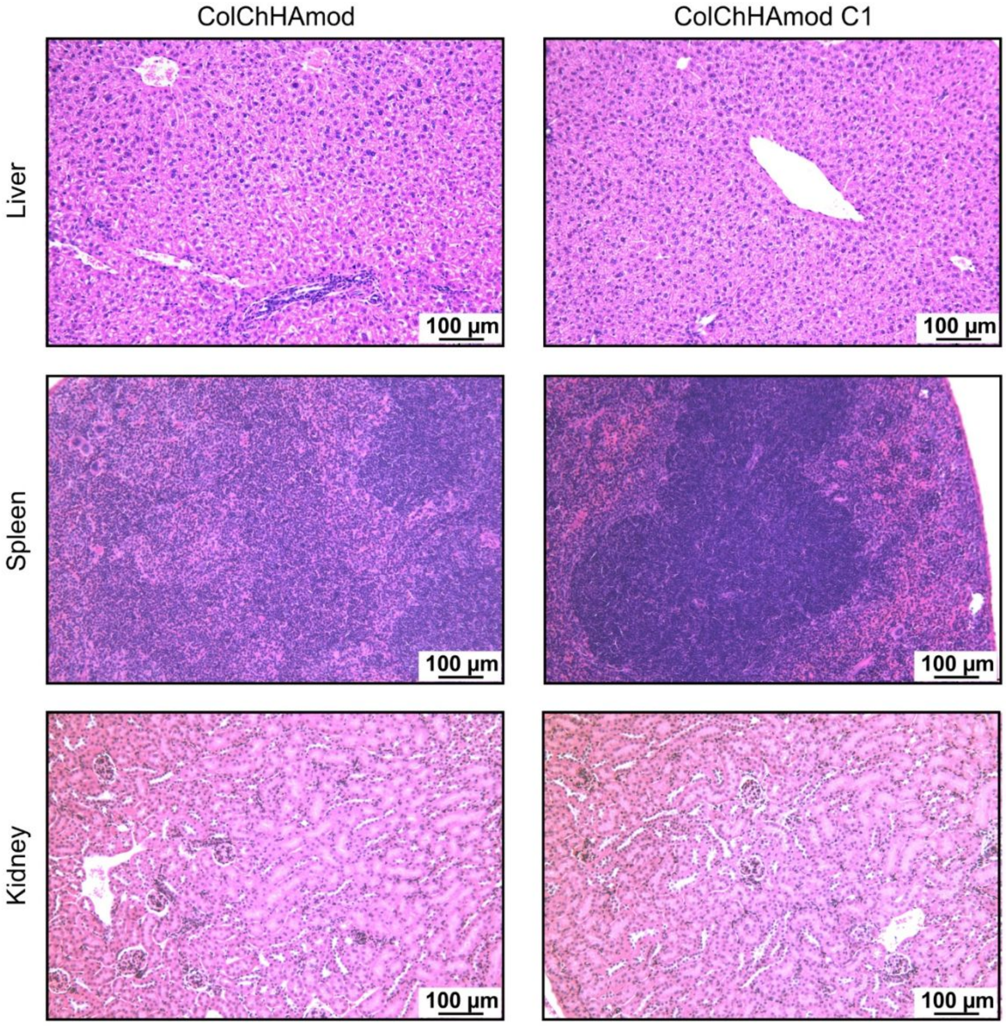

Figure S6. Histological examination of the liver, spleen, and kidney isolated from animals after 60 days post materials injection.

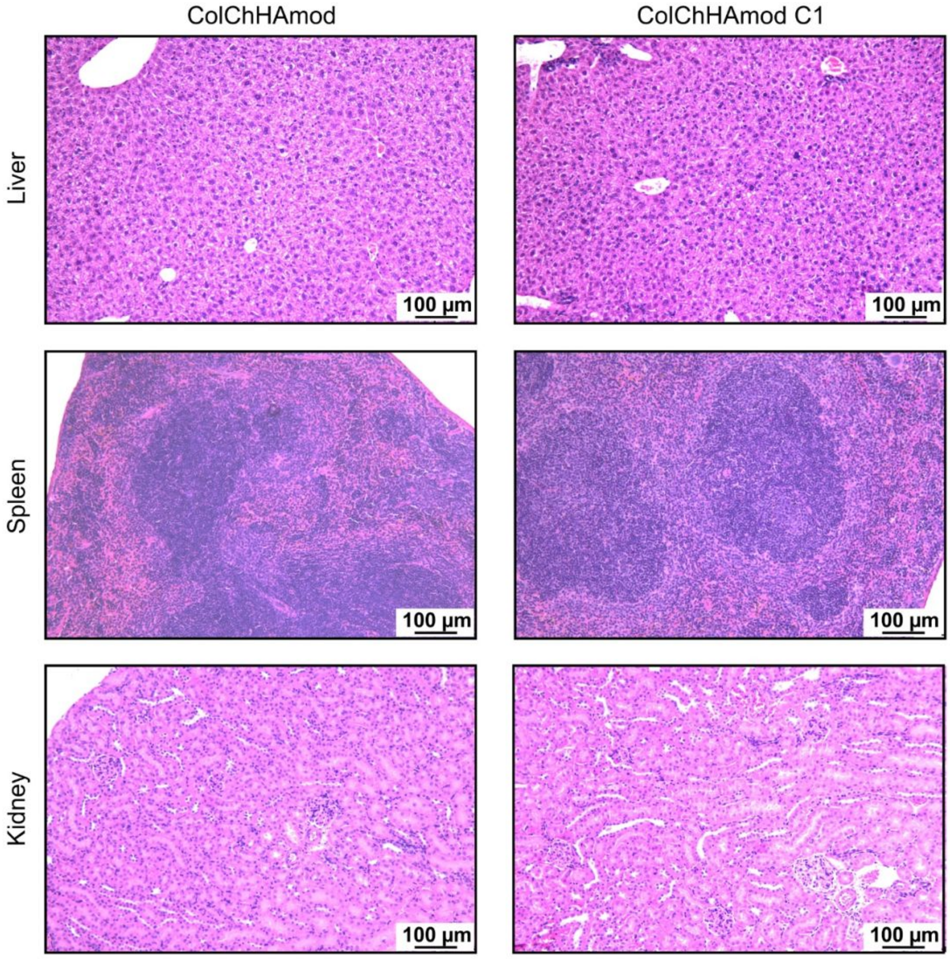

References:

(1) Gilarska, A.; Lewandowska-Łańcucka, J.; Guzdek-Zając, K.; Karewicz, A.; Horak, W.; Wójcik, K.; Nowakowska, M. Bioactive yet Antimicrobial Structurally Stable Collagen / Chitosan / Lysine Functionalized Hyaluronic Acid – Based Injectable Hydrogels for Potential Bone Tissue Engineering Applications. *Inter. J. Biol. Macromol*. **2020**, *155*, 938-950.
